# MCM2-7 loading-dependent ORC release ensures genome-wide origin licensing

**DOI:** 10.1038/s41467-024-51538-9

**Published:** 2024-08-24

**Authors:** L. Maximilian Reuter, Sanjay P. Khadayate, Audrey Mossler, Korbinian Liebl, Sarah V. Faull, Mohammad M. Karimi, Christian Speck

**Affiliations:** 1 DNA Replication Group, Institute of Clinical Sciences, Faculty of Medicine, Imperial College London, London, United Kingdom; 2 MRC London Institute of Medical Sciences (LMS), London, United Kingdom; 3 Department of Chemistry, Chicago Center for Theoretical Chemistry, Institute for Biophysical Dynamics, and James Franck Institute, The University of Chicago, Chicago, IL USA; 4 Comprehensive Cancer Centre, School of Cancer & Pharmaceutical Sciences, Faculty of Life Sciences & Medicine, King’s College London, London, United Kingdom; 5 Present Address: Institute of Molecular Biology (IMB) gGmbH, Ackermannweg 4, Mainz, Germany

**Keywords:** DNA, Origin selection

## Abstract

Origin recognition complex (ORC)-dependent loading of the replicative helicase MCM2-7 onto replication origins in G1-phase forms the basis of replication fork establishment in S-phase. However, how ORC and MCM2-7 facilitate genome-wide DNA licensing is not fully understood. Mapping the molecular footprints of budding yeast ORC and MCM2-7 genome-wide, we discovered that MCM2-7 loading is associated with ORC release from origins and redistribution to non-origin sites. Our bioinformatic analysis revealed that origins are compact units, where a single MCM2-7 double hexamer blocks repetitive loading through steric ORC binding site occlusion. Analyses of A-elements and an improved B2-element consensus motif uncovered that DNA shape, DNA flexibility, and the correct, face-to-face spacing of the two DNA elements are hallmarks of ORC-binding and efficient helicase loading sites. Thus, our work identified fundamental principles for MCM2-7 helicase loading that explain how origin licensing is realised across the genome.

## Abstract (web summary)

Correct loading of the MCM2-7 helicase is crucial for DNA replication and cell cycle progression. Here, the authors used high-resolution genomics to demonstrate how ORC is displaced from origins, which serves as a mechanism for distributive MCM loading onto DNA.

## Introduction

Replication origins serve as central sites for origin recognition, helicase loading, initial DNA unwinding, and replication fork establishment. The accurate regulation of these events is central to genome stability^1,2^. *Saccharomyces cerevisiae* contains about 350 confirmed replication origins, also termed autonomous replication sequences (*ARS*). These contain genetically defined A-, B1-, and B2-elements^3,4^. The A- and B1-elements function as the primary binding sites of the origin recognition complex (ORC)^5,6^, while the B2-element is poorly defined at a sequence level and has been proposed to act both as a secondary ORC binding site^7,8^ and as a DNA unwinding element^9^. During early G1-phase, ORC, with the help of Cdc6 and Cdt1, loads two copies of the hetero-hexameric MCM2-7 helicase onto double-stranded DNA (dsDNA), a process termed DNA licensing^10^. As MCM2-7 represents the catalytic core of the replicative helicase, it is essential that DNA licensing occurs evenly throughout the genome, otherwise sections of DNA would be left unreplicated^11^. Once the MCM2-7 double hexamer (DH) forms on DNA, it is initially inactive for DNA unwinding^12^. During the G1-S-phase transition, S-phase specific kinases and a series of replication factors activate the MCM2-7 double hexamer to promote the formation of bidirectional replication forks^6,10,13,14^.

Although helicase loading has been investigated in vitro and in vivo^15–17^, it is still unclear how specific origin sequences are utilised for helicase loading in vivo. Indeed, the precise genomic binding sites of ORC and MCM2-7 are not fully understood. Therefore, we do not know whether helicase loading follows a “distributive” loading mechanism, where maximal one MCM2-7 DH is loaded at a high-efficiency origin, or a “processive” mechanism, where repetitive MCM2-7 loading occurs at high-efficiency origins. Uncovering the binding sites of ORC and MCM2-7 at near base-pair resolution has the potential to reveal how the genome can be licensed for DNA replication.

Using high-resolution ChIP-Exo^18^ we show that ORC and MCM2-7 have overlapping origin binding sites, which renders DNA licensing a self-limiting process that ensures distributive helicase loading along the genome. Moreover, our bioinformatic analysis uncovered a specific B2 sequence motif. Interestingly, we found that the A- and B2-elements demarcate the MCM2-7 DH location at replication origins, revealing that DHs are generally immobile following loading and that origins adopt a very compact organisation in vivo. As such, our work explains how replication factors utilise replication origins to promote and regulate genome-wide helicase loading.

## Results

### Identification of the ORC and MCM2-7 binding sites at base pair resolution

**Fig. 1:**
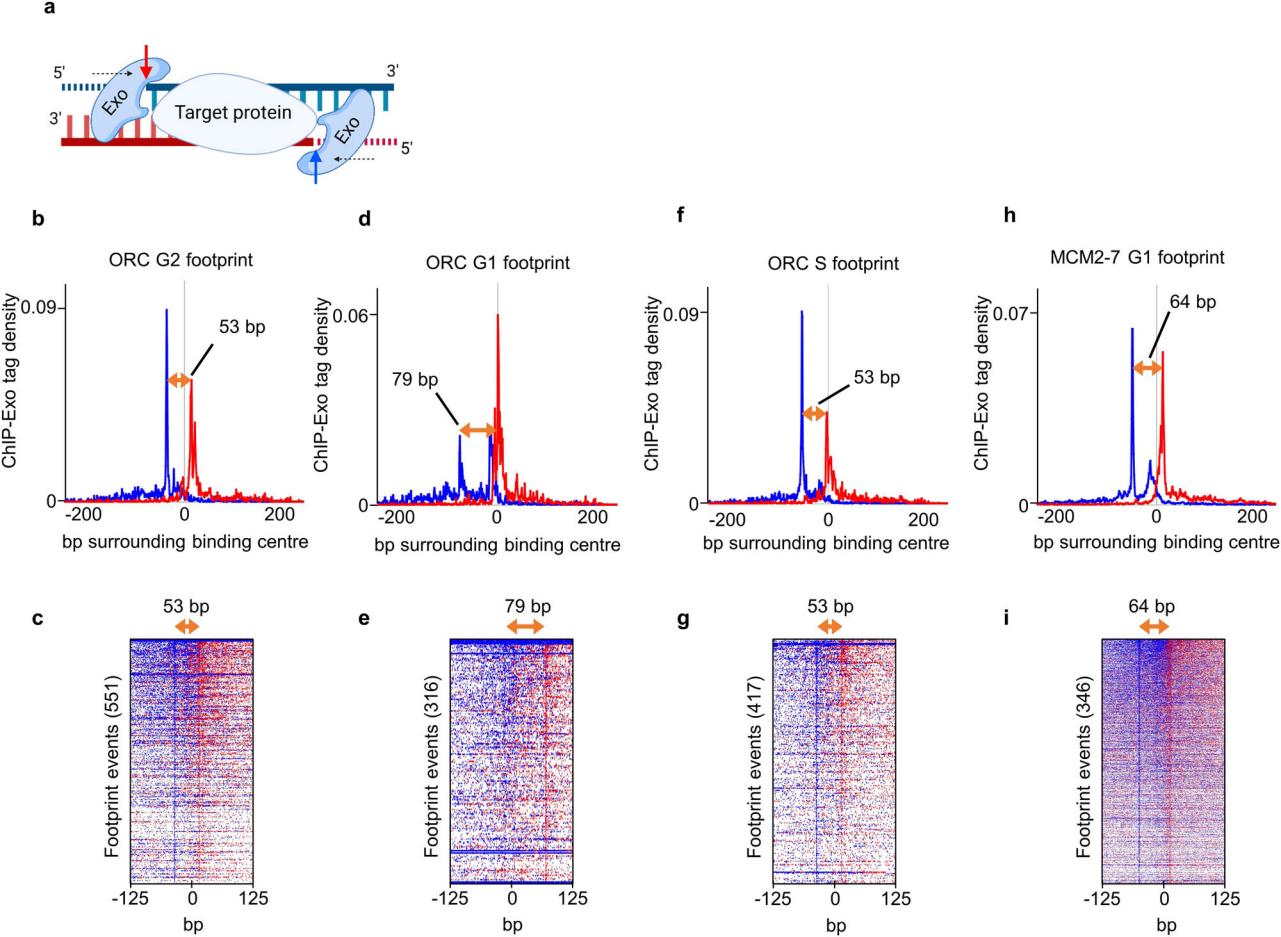
ChIP-Exo identifies precise ORC and MCM2-7 binding sites at origins in *S. cerevisiae.* **a** Lambda exonuclease digestion yields molecular footprint specificity in ChIP-Exo 5.0. Cross-linked and sheared chromatin is immunoprecipitated via the tagged protein. After washing, protruding DNA is digested with Lambda exonuclease and unique molecular identifiers are ligated before the DNA is eluted and libraries are amplified. **b**, **d**, **f**, and **h** Composite plots of ChIP-Exo 5.0 tag distribution patterns (forward strand in blue and reverse strand in red) of (**b**) Orc2 from G2-arrested cells, (**d**) Orc2 from G1-arrested cells, (**f**) Orc2 in S-phase (24 min post alpha-factor release), and (**h**) MCM2-7 double hexamers (Mcm4) from G1-arrested cells ±250 bp. The individual MCM2-7 DH and ORC footprint sizes are indicated (orange arrows). **c**, **e**, **g**, and **i** Representative heat maps of ChIP-Exo 5.0 tag enrichments, sorted by decreasing signal, of the footprint events shown in **b**, **d**, **f**, and **h** of Orc2 and Mcm4 ± 125 bp. The individual MCM2-7 DH and ORC footprint sizes are indicated (orange arrows). **a** created with BioRender.com released under a Creative Commons Attribution-NonCommercial-NoDerivs 4.0 International license. Source data is provided as a Source Data file.

It is well established that in G2-phase only ORC is bound to the origin, while in G1-phase MCM2-7 helicase loading occurs^19^. On the other hand, whether the MCM2-7 DH binds to specific origin sequences and how MCM2-7 loading transforms the origin organisation is only partially understood. To address these questions, we generated genomically FLAG-tagged Orc2 and Mcm4 yeast cells and employed ChIP-Exo to study protein-DNA interactions. The genome-wide footprints generated by ChIP-Exo are compact, as the method uses an exo-nuclease to define the edge of the protein-DNA interaction site at near base-pair resolution^18,20^ (Fig. 1). Thus, our approach identified the location of ORC in G2-, G1-, and S-phase and MCM2-7 DHs in G1-phase at unprecedented resolution.

ChIP-Exo mixture model (ChExMix)^21^ analysis of 346 characterised replication origins showed that ORC in G2-phase covers, on average, a 53 bp stretch of DNA (Fig. 1b and c), which increased to 79 bp in G1-phase (Fig. 1d and e) and decreased to 53 bp in S-phase (Fig. 1f and g). The footprints’ sizes and locations are consistent with previous in vitro and in vivo observations at individual origins^22–24^. MCM2-7 DHs produced a footprint of 64 bp, consistent with a recent genomic study and previous cryo-EM studies that visualised the MCM2-7 DH on DNA (Fig. 1h and i)^25–28^. To understand the distribution of the helicase across the origin, we measured the average normalised MCM2-7 DH occupancy by read depth in an 800 bp fragment surrounding each origin boundary. We found that 78% of the read counts were localised in a narrow 60 bp window (Supplementary Fig. S1). Consistently, we identified that ~41.5% of origins (143/346) exhibit isolated, sharp, and single peaks (Supplementary Fig. S1a), ~25.5% (88/346) exhibit several peaks with a prominent single peak (Supplementary Fig. S1b, lower panel), and ~33% (115/346) showed multiple ( ≥ 2), often equally strong MCM2-7 DH peaks per origin (Supplementary Fig. S1b, upper panel). On the other hand, the multi-MCM2-7 DH origins frequently show multiple ORC peaks ( ~ 82%), with only a minority harbouring single ORC peaks ( ~ 18%, Supplementary Fig. S1b and c). This suggests that single MCM2-7 DHs become loaded at replication origins while multi-MCM2-7 peaks are less frequent and may rather reflect the differential use of ORC binding sites across cell populations.

**Fig. 2:**
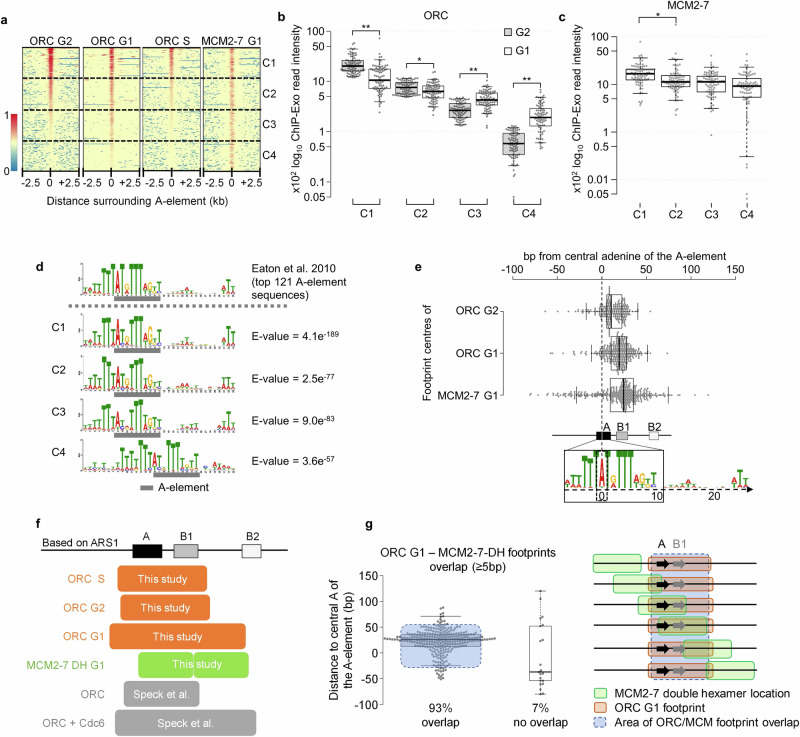
ORC and MCM2-7 binding sites overlap at origins. **a** ChIP-Exo 5.0-derived heat maps of Mcm4 and Orc2 binding in G1-, G2-, and S-phase at 346 origins. Centred are A-elements ± 2.5 kbp, sorted by ORC signal intensity in G2-phase. **b** Quantification of ORC binding in clusters C1-C4 from (**a**). Individual values, as well as the median, 1st, and 3rd quartiles, are shown. Whiskers extend to 5th and 95th percentile. Significance levels were calculated using two-sided Wilcoxon Signed-rank tests from 81 origins (C1-C3) and 80 origins (C4) (*: *p* < 0.0001, **: *p* < 0.00001). **c** Quantification of MCM2-7 DH binding in clusters C1-C4 from (**a**). Data representation and statistics as in (**b**). **d** ORC binding intensity correlates with A-element similarity. Comparison of the standard A-element sequence^62^ with the identified motifs derived from ORC heat map clustering in (**a**). The standard A-element locations are highlighted (grey bar). **e** MCM2-7 DH and ORC binding sites overlap at origins. Distance calculations from the central adenine (dotted line) of the A-element sequences to the binding centre of MCM2-7 (*n* = 329), ORC in G1-phase (*n* = 301) and ORC in G2-phase (*n* = 229) are depicted. The locations of conserved origin DNA elements are based on *ARS1* and were aligned with the consensus A-element sequence. Individual distances (dots) as well as the median, 1st, and 3rd quartiles are shown. Whiskers extend to < 1.5x IQR from 1st and 3rd quartiles. **f** Graphic overview of in vivo ORC footprints in G1-phase (79 bp) and G2-phase (53 bp) and MCM2-7 DH footprints in G1-phase (64 bp) relative to the *ARS1* origin and comparison with published in vitro footprints^23^. **g** Characterisation of MCM2-7 DH and ORC overlap in G1-phase. Distances from the central adenine of the A-element (0 bp) to the binding centres of individual MCM2-7 DHs are shown (*n* = 301). 93% of DHs overlap with the corresponding ORC footprint in G1-phase. Individual distances (dots) as well as the median, 1st, and 3rd quartiles are shown. Whiskers extend to < 1.5x IQR from 1st and 3rd quartiles. The area where 93% of ORC and MCM2-7 binding sites overlap is indicated (blue box, 5th to 95th percentile). Schematic overview of MCM2-7 DH (green) and ORC (orange) locations on *ARSs* with overlap (blue). For **a**–**g** source data is provided as a Source Data file.

Next, we studied the location of ORC in G2- and G1-phase. Comparing all origin binding events of ORC revealed differential binding in G2- and G1-phase, similar to previous observations (Fig. 2a)^17^. Based on the G2-phase ORC binding we classified origins into four groups (clusters C1-C4) each representing ~25% of origins (Fig. 2a–c). We then analysed the underlying sequences of clusters C1-C4 and found that cluster C1 contains origin sequences that are highly similar to the known A-element consensus sequence, while clusters C2-C4 displayed decreasing degrees of similarity (Fig. 2d). Consistent with the idea that budding yeast ORC has DNA sequence specificity, we suggest that a less conserved A-element results in reduced ORC interaction when compared to an origin containing a highly conserved A-element^22,29^ and that this ultimately correlates with replication timing (Supplementary Fig. S2a). Interestingly, cluster C1 was enriched for origins bound near telomeres and centromeres (Supplementary Fig. S2b), suggesting that several origins in specialised chromatin are highly occupied by ORC.

Surprisingly, cluster C1 and C2 sites with high ORC occupancy in G2-phase showed a 48% and 17% reduction in Orc2 occupancy in G1-phase, respectively, while cluster C3 and C4 sites with low Orc2 in G2-phase displayed a 62% and 231% increase in Orc2 binding in G1-phase (Supplementary Fig. 2a, b, and Supplementary Fig. S2a and c). Thus, the data indicate that ORC becomes released from C1 and C2 sites during the G2- to G1-phase transition and accumulates in G1-phase at C3 and C4 sites. Finally, when analysing MCM2−7 DH binding, we found that C1 sites displayed the most robust Mcm4 occupancy while clusters C2, C3, and C4 showed reduced occupancies (C1 to C2: −33%, C2 to C3 and C4 −19%, Fig. 2c). Hence, although clusters C1-C4 displayed pronounced differences in ORC occupancy (Fig. 2a and b), MCM2-7 loading varied to a lesser degree, in line with the idea of even distribution of origin licensing across the genome (Fig. 2c). Finally, origins that display a stronger reduction in ORC occupancy during the G2- to G1-phase transition displayed increased MCM2-7 loading (Fig. 2c and S2d). Thus, sites that show strong ORC displacement are frequently sites of efficient origin licensing, a trend that was not observed for C3 and C4 sites (Figs. 2c and S2d). In summary, we conclude that ORC shows high affinity binding to a subset of origin in G2 phase and becomes displaced from these high-efficiency origins during the G2-G1 transition.

Next, we wondered where ORC and MCM2-7 DH binding sites were located within origin sequences. Therefore, we analysed the origin binding sites of ORC and MCM2-7 more closely by plotting the location of the ORC and MCM2-7 DH footprint centres in G2- and G1-phase (Fig. 2e). This analysis shows that ORC in G2-phase is centred across the A- and B1-elements. In G1-phase, when ORC is known to interact with Cdc6, the ORC footprint expands^19,24,30,31^. Binding centres are located predominantly near the B1-element, similar to the in vitro ORC-Cdc6 binding^23^ (Fig. 2f, Supplementary Data 1). Once cells have committed to S-phase, this footprint shrinks again to the G2-phase footprint, indicative of Cdc6 degradation^32^ and licensing incompetence. Importantly, 93% of the MCM2-7 footprint centres were located in a confined area that overlapped with the ORC footprint, as measured for individual origins (Fig. 2g). Conversely, only 7% of MCM2-7 DH binding sites did not overlap with the 79 bp ORC G1-phase footprint. In summary, the data show that MCM2-7 DH becomes loaded across the primary ORC binding site and highlights that the MCM2-7 DH assumes a fixed position at origins (Fig. 2e and g).

### ORC becomes dynamically redistributed in G1-phase

**Fig. 3:**
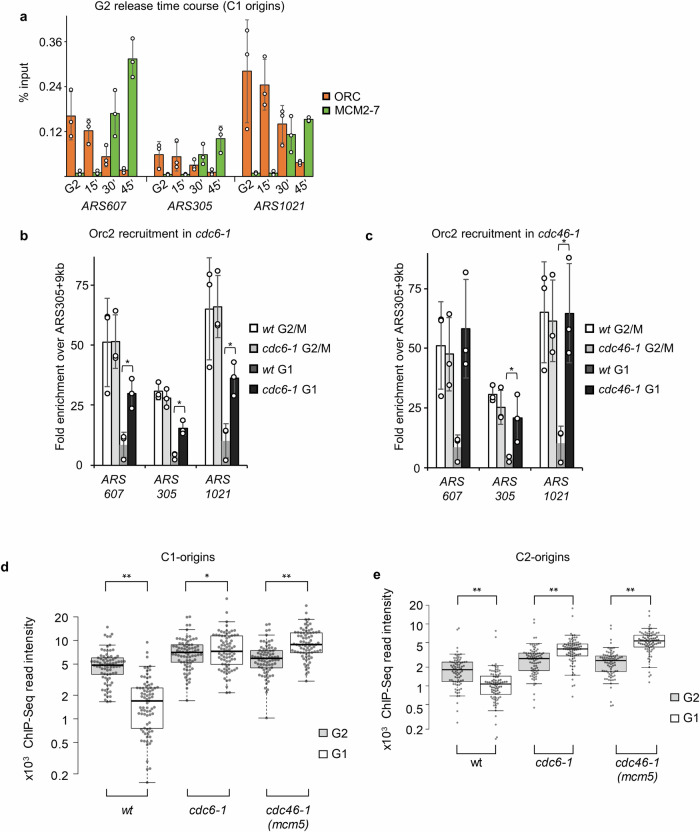
DNA licensing causes ORC release in G1-phase of the cell cycle. **a** MCM2-7 DHs and ORC occupy the same DNA sequentially. ChIP-qPCR analysis of Orc2 or Mcm4 at early origins (*ARS1021*, *ARS607*, and *ARS305*). G2-phase release time course showing ORC loss and MCM2-7 loading upon transition from G2- into G1-phase using a conditional *cdc20* mutant. **b**, **c** Reduced DNA licensing stabilises ORC at origins. ChIP-qPCR analysis of Orc2 at selected early activating origins of G2/M- and G1-phase arrested for wt and *cdc6-1* cells (**b**), or *cdc46-1* (*mcm5*) ts-mutant cells (**c**) at the non-permissive temperature (37 °C). Occupancies are fold enrichments over non-bound DNA close to *ARS305* (*ARS305* + 9 kbp). qPCR data are the average and standard deviation derived from three biological replicates. Significance levels were calculated using two-sided Student’s t-tests (*: *p* < 0.05). **d** and **e** Inhibiting DNA licensing stabilises ORC at origins. ChIP-Seq-derived, genome-wide quantification of ORC binding (Orc2) in wt, *cdc6-1*, and *cdc46-1* (*mcm5*) ts-mutant cells in G2- and G1-phase at the non-permissive temperature (37 °C). Individual values of C1- (**d**) and C2-clustered origins (**e**) as well as the median, 1st, and 3rd quartiles are shown. Whiskers extend to the 5th and 95th percentile. Significance levels were calculated using two-sided Wilcoxon Signed-rank tests (C1 (*n* = 78) and C2 (*n* = 80)) (*: *p* < 0.01, **: *p* < 0.0000000001). For (a-e), source data is provided as a Source Data file.

As the MCM2-7 DH and ORC cannot occupy the same DNA sequence simultaneously, we hypothesised that ORC binds to origins and subsequently becomes displaced during helicase loading, resulting in MCM2-7 DHs occupying the primary ORC binding sites. To test this model, we performed a time course analysis of ORC and MCM2-7 recruitment to selected replication origins during the G2/M-G1-phase transition (Fig. 3a). We found that Orc2 is initially bound to cluster C1 replication origins but was greatly reduced at the 30 and 45 min time points. Importantly, we observed increased Mcm4 recruitment at the same time points when ORC was released, suggesting that DNA licensing is responsible for ORC release. To test this concept directly, we asked whether inhibition of DNA licensing during the G2/M-G1-phase transition would reduce ORC displacement. We employed *cdc6-1* and *cdc46-1* (*mcm5*) temperature-sensitive alleles, arrested cells in G2/M- or G1-phase and observed that ORC displacement was significantly reduced at non-permissive conditions in G1-phase (Fig. 3b and c). A genome-wide analysis by ChIP-Seq further corroborated that ORC release from class C1 and C2 origins was blocked in the context of c*dc6-1* and *cdc46-1* (*mcm5*) when DNA licensing is hindered (Fig. 3d and e). Thus, our data demonstrate that ORC is displaced from efficient origins in an MCM2-7 DH loading-dependent manner, and ORC rebinding to these origins is suppressed.

### Non-origin binding sequences serve as an ORC sink in G1-phase

**Fig. 4:**
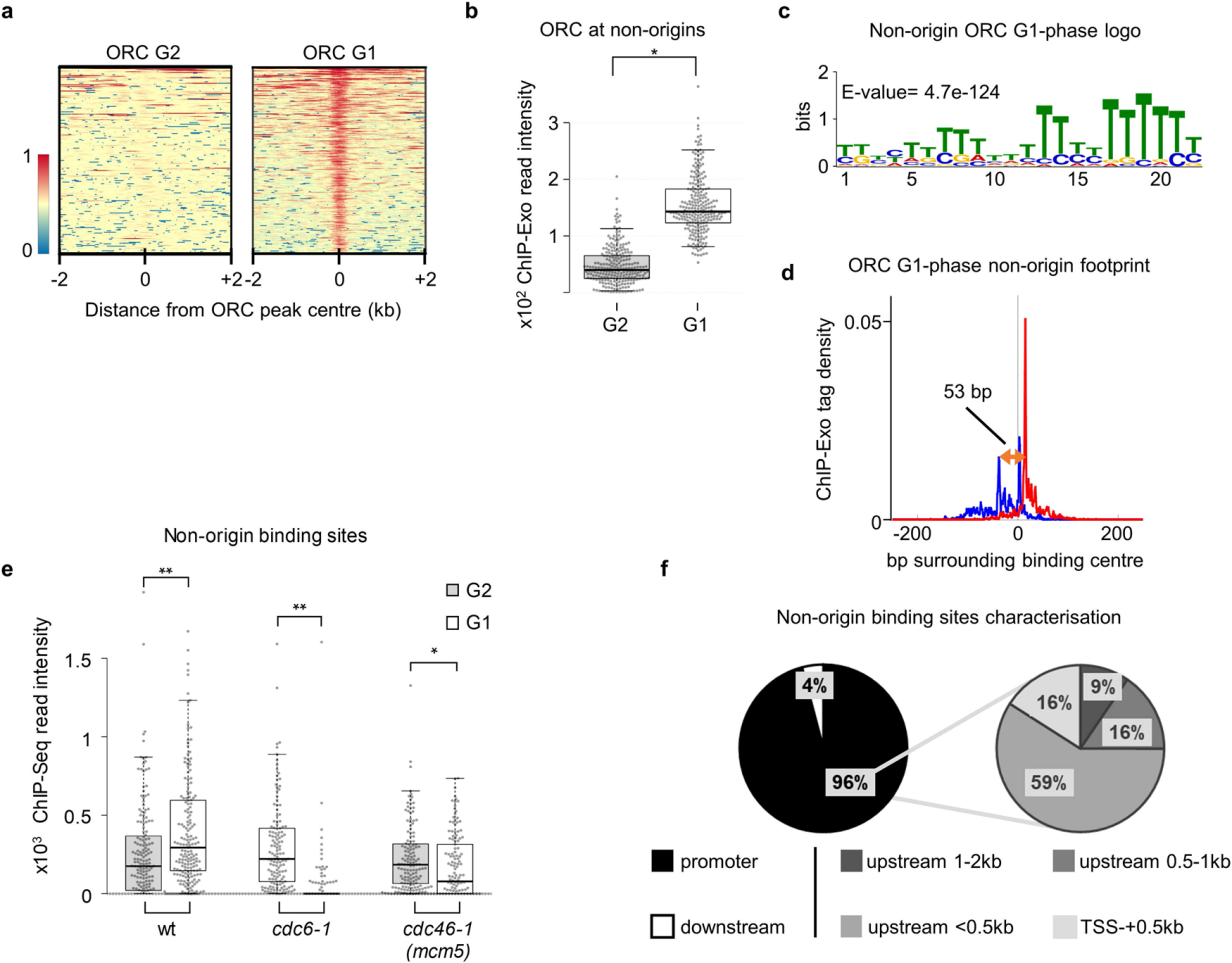
ORC redistributes from origins to non-origin binding sites in G1-phase. **a** ChIP-Exo 5.0-derived heat maps of Orc2 (ORC) in G1- and G2-phase at non-origin binding sites (*n* = 242). Centred are peak signal intensities ±2 kbp, sorted by Orc2 signal intensity in G1-phase. **b** Quantification of ORC signals at non-origins binding sites in G2- (*n* = 235) and G1-phase (*n* = 242). Individual values, as well as the median, 1st, and 3rd quartiles, are shown. Whiskers extend to the 5th and 95th percentile. Significance levels were calculated using two-sided Student’s t-tests (*: *p* < 0.0000000001). **c** Motif finding at non-origin binding sites retrieved a pyrimidine-rich motif as an alternative ORC binding motif (57% of all sites). **d** Composite plot of ChIP-Exo 5.0 tag distribution patterns (forward strand in blue and reverse strand in red) of Orc2 from G1-phase arrested cells ± 250 bp at non-origin binding sites. The individual ORC footprint size is indicated (orange arrow). **e** Inhibiting DNA licensing reduces ORC occupancy at non-origin binding sites. ChIP-Seq-derived, genome-wide quantification of Orc2 binding (ORC) in wt, *cdc6*-1, and *cdc46*-1 (*mcm5*) ts-mutant cells in G2- and G1-phase at the non-permissive temperature (37 °C). Individual values, as well as the median, 1st, and 3rd quartiles, are shown. Whiskers extend to the 5th and 95th percentile. Significance levels were calculated using two-sided Wilcoxon Signed-rank tests from origins (wt (*n* = 171), *cdc6-1* (*n* = 136), and *cdc46-1* (*n* = 138) (*: *p* < 0.01, **: *p* < 0.00001). **f** ORC peaks at non-origin binding sites localise to promoters. Pie charts highlighting the binding region of non-origin binding sites of ORC in G1-phase. For (**a**–**c**) and (**e**–**f**) source data is provided as a Source Data file.

If ORC becomes released from replication origins in G1-phase due to DNA licensing and rebinding to the same origin is not possible, one would predict that in turn, ORC should accumulate at non-origin sites. To test this hypothesis, we examined our ORC ChIP-Exo data for ORC binding to sites outside of origins in G1-phase. This analysis identified ORC binding to 242 non-origin sites in G1-phase, with little to no binding in G2-phase (Fig. 4a and b, Fig. S3a and b, Supplementary Data 1). This was confirmed by ChIP-qPCR analyses at selected non-origin sites (Fig. S3c and d). Generally, Orc2 read intensities at non-origin DNA sites were comparable to C4 origin sites (Fig. S3e). Furthermore, sequence motif analysis of the non-origin bound ORC sites identified a pyrimidine-rich binding motif with reduced sequence specificity than the consensus ORC binding site (Fig. 4c). Interestingly, ORC’s footprint size at these non-origin sites was 53 bp, indicating a binding mode similar to the licensing incompetent ORC that binds to DNA in G2- or S-phase (Fig. 4d, compare with Fig. 1b and f). Consistent with that, we did not observe MCM2-7 signals at these sites (Fig. S3b). To understand whether the binding of ORC to non-origin sites was linked to ORC release from cluster C1 sites, we analysed the *cdc6-1* and *cdc46-1* ChIP-seq data for non-origin sites (Fig. 4e). Indeed, when DNA licensing was blocked and ORC release does not occur (Fig. 3), binding of ORC to non-origin sites was blocked (Fig. 4e). Analysis of all non-origin binding sites found ORC predominantly bound to promoters close to origins, mostly within 1 kbp upstream of the transcription start site (TSS) (Fig. S3f and Fig. 4f). Specifically, 89% of non-origin binding sites were within 380 bp of the TSS and 55% were near TFIIB-binding motifs. In comparison, ORC bound to origins was less enriched for TSS sites, in line with the fact that origins are intergenic features (Fig. S3g). The small ORC footprint size at non-origin sites strongly suggests that Cdc6 is not recruited to these sites. Together with the absence of MCM2-7, the data highlights that ORC interacts in G1-phase with a new set of non-origin DNA sequences, which are T-rich and characterised by open chromatin.

### The role of the B2-element in DNA licensing

Recent single-molecule experiments revealed that a sole ORC molecule can participate in the loading of the first and second MCM2-7 hexamer^8^. Initially, ORC binds to its primary binding site, the A-element, where it loads the first MCM2-7 hexamer. The second MCM2-7 hexamer loading event benefits from a specific ORC-MCM2-7 interaction^15^, which allows ORC to flip and transition from its primary DNA binding site to a secondary binding site.

However, ORC’s secondary DNA binding site at replication origins remains ill-defined. One possibility is that the B2-element could serve as this additional ORC binding site^8,15^. So far, very few B2-elements have been experimentally defined^33^. Mutational analyses of the B2 element highlighted that it is important for DNA replication, but due to the poor sequence conservation, it was difficult to explore it in more detail^29,33^. Indeed, as the experimentally identified B2-elements differed widely in sequence, length, and location, it proved challenging to define a clear-cut motif, which predicts not only the correct sequence but also the correct location and orientation of the B2-element^7,33,34^. Thus, we wondered whether our MCM2-7 ChIP-Exo data could reveal insights into B2-element sequence, location, and function.

As ORC binds preferentially to T-rich sequences^22^, we initially investigated the thymidine composition near MCM2-7 binding sites. Aligning DNA sequences bound by the most or least abundant MCM2-7 origins (Fig. S4a) revealed the presence of an AT-skew across the DH binding site. Moreover, when sorting the MCM2-7 DH-covered sequences by their replication time^35,36^, we observed that early origins, which load MCM2-7 efficiently, display a more uniform AT-skew, a more canonical A-element and a clear bias towards an A-rich sequence in the proximity of the putative B2-element site (Fig. S4b, upper panels). This suggests that the AT-skew will help to position ORC in a head-to-head configuration (Fig. S4C). Moreover, this DNA sequence arrangement will limit helicase loading specifically to the AT-skew region, which in turn generates specificity. However, aligning these origin sequences by the A-element failed to identify a clear-cut B2 motif (Fig. S4b, lower panel).

**Fig. 5:**
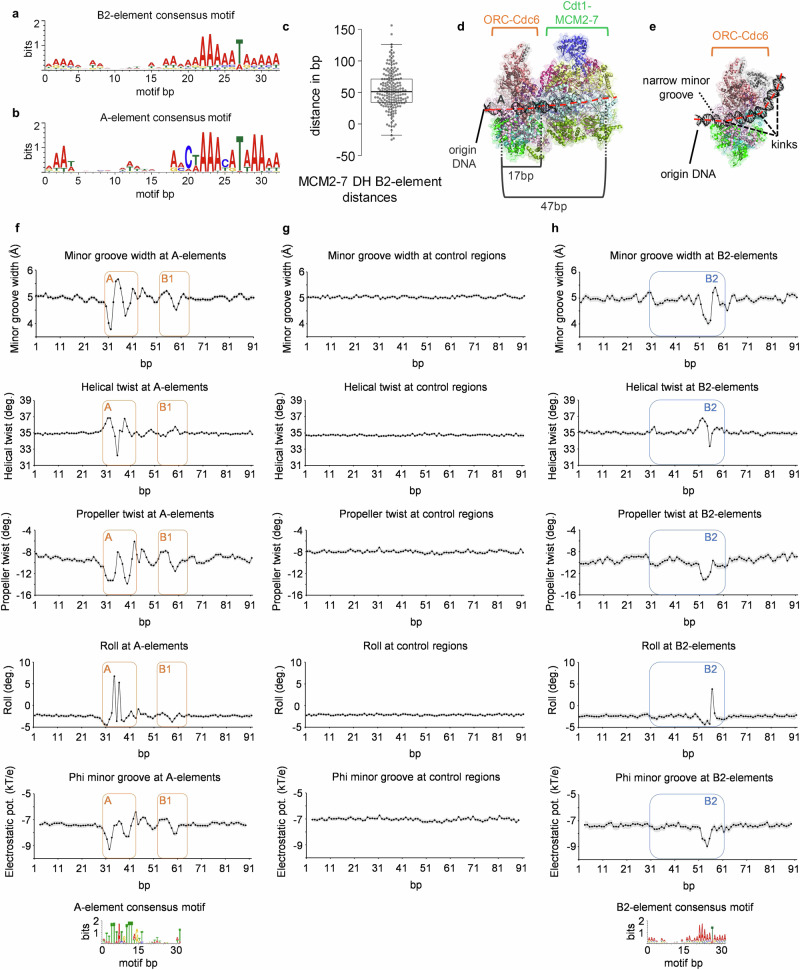
Genome-wide search for a B2 sequence motif and its properties. The B2-element resembles the characteristics of an inverted A-element. The identified B2-element consensus motif (**a**) is shown in comparison to the reverse-complement consensus motif of the A-element (**b**) as in Fig. 2d. **c** Distance calculation from the centre of MCM2-7 DHs to the corresponding, correctly-orientated B2-element in G1-phase (*n* = 181). Individual distances (dots) as well as the median, 1st, and 3rd quartiles are shown. Whiskers extend to <1.5x IQR from 1st and 3rd quartiles. **d** Three-dimensional view of the OCCM complex (modified from PDB: 6WGI), highlighting the path of dsDNA (red, *ARS416* (*ARS1*) origin) through ORC-Cdc6 and the first loaded Cdt1-MCM2-7 complex. Estimated distances covered by ORC and MCM2-7 are indicated. **e** Three-dimensional view of the ORC-Cdc6-85 bp DNA complex (modified from PDB: 7MCA), highlighting the path of dsDNA (red) through ORC-Cdc6. *ARS416* (*ARS1*) sequence is shown with successive bending of the DNA illustrated by the dashed lines (black). **f**, **g** and **h** DNA is deformed at A- and B2-elements *in S. cerevisiae*. DNA shape changes at A-elements (**f**), control sequences (500 bp downstream of A-elements) (**g**), and the newly identified B2-elements (**h**). Changes in minor groove width, helical twist, propeller twist, roll (GB shape^48^), and electrostatic potential (phi; an approximation for steric impact on groove geomety^47^) are depicted ± 30 bp surrounding the respective DNA element. Individual dots represent the median deformation at a given position with the surrounding 95% confidence interval shown in grey. A- and B1-elements are highlighted by orange boxes, and the B2-element by blue boxes with a representation of the underlying consensus motifs. For **a**–**c** and **f**–**h**, source data is provided as a Source Data file.

Thus, we wondered whether B2 sequences may have a variable distance to the A-element. We searched for a potential B2-element sequence next to the MCM2-7 double-hexamer ChIP-Exo footprints, focussed on AT-rich sequences that are known to support ORC-Cdc6 complex formation^37^, and took advantage of experimentally defined B2 sequences^7,33^. In an iterative process, we identified a DNA sequence that predicts a B2-element motif at most replication origins. This motif fits well with low-throughput genetically determined B2 sequences^29,33,38–41^ and predicts B2 sequences with much higher confidence levels than a previous study^33^ (Figs. 5a and S5, Supplementary Data 1). To our surprise, the motif had similarities in sequence and composition with the A-element (reverse complement due to opposite strand direction) but was completely devoid of the conserved cytidine nucleotides and had, in part, an altered AT content (Figs. 5b and S5). The genome-wide analysis identified B2 motifs within 150 bp of the A-element in 224 of 308 origins, and 181 of 224 B2 motifs were in the correct orientation to support head-to-head MCM2-7 double-hexamer formation (Fig. S6a). At 43 origins, an incorrectly orientated B2-element was identified and 84 origins did not show a B2 motif (Fig. S6a). 114 of 181 origins contained a single B2 motif and 67 origins showed 2–5 sequential matches to the motif (Fig. S6b). The median distance between the centre of MCM2-7 DH and the first B2-element was 51 bp (Fig. 5c), and 19 bp when measured from the edge of the double-hexamer footprint to the conserved T residue. We note that this distance is compatible with ORC binding to the B2-element and promoting MCM2-7 DH loading since the OCCM structure identified a distance of 17 bp between the conserved thymidine and the edge of the MCM2-7 hexamer^42^ (Fig. 5d). Thus, the data suggest that the newly identified B2-element is optimally positioned to support ORC-dependent MCM2-7 DH formation. Moreover, the proximity of the MCM2-7 complex to the B2-motif highlights that the loaded helicase remains predominantly static on DNA once the DNA licensing reaction has concluded.

Although the B2 sequence motif is not as well defined as the A-element, we wondered whether the underlying B2 sequences are conserved among seven *Saccharomyces* species (*S. cerevisiae*, *S. paradoxus*, *S. mikatae*, *S. kudriavzevii*, *S. bayanus*, *S. castelli*, and *S. kluyveri)*^43^. While our analysis showed a clear sequence conservation for the A-element, we did not observe this for the B2-element (Fig. S6c and d), highlighting that the DNA sequence of the B2-element at individual origins is not conserved across various *Saccharomyces* species.

### The B2-element shares structural features with the A-element

It has been shown that the origin DNA within the ORC-DNA and ORC-Cdc6-DNA cryo-EM structures is strongly kinked, *i.e*. the minor groove is deformed^44,45^ (Fig. 5e), and that *Drosophila* ORC binding sites share an unusual electrostatic potential^46^. Thus, to investigate whether budding yeast ORC DNA binding sequences adopt unusual features, we predicted DNA structural properties of the A-element. We focussed on origins that contained both a ChIP-Exo ORC footprint and a predicted B2-element. We studied minor groove width, propeller twist, roll, helix twist, and electrostatic potential in the minor groove of the well-established A-elements and compared this to a downstream control region^47,48^. The analysis suggests that the primary ORC binding DNA sequences, in contrast to the control regions, contain an intrinsic, unusual shape even in the absence of its protein binding partner (Fig. 5f and g). We identified a structural signature at the A-element, which is characterised by a narrow minor groove, a wider minor groove that is followed by another narrow minor groove. This pattern is reflected in major changes to the propeller twist, roll, helix twist, and electrostatic potential. In addition, a second deformation was found at the B1-element, which coincides with a stretch of A/T-residues. Indeed, in at least three-quarters of the origins analysed, a substantial deviation in DNA shape was observed (Fig. S6E). Next, we asked whether the discovered B2 motif is also associated with any changes in the DNA shape and related features (Fig. 5h). We identified that the first dip in minor groove width was reduced when compared to the A-element, but the second dip was wider. These differences are equally reflected by helical twist, propeller twist, roll, and electrostatic potential. A B1-like second deformation can also be seen in the context of the B2-element. Again, we identified that at least three-quarters of the B2-element sequences showed a substantial deviation in DNA shape and electrostatic potential (Fig. S6e). Origins that contain multiple B2-elements presented a change in the minor groove width for each B2-element, but this was most pronounced for the closest B2-element (Fig. S6f). Thus, we can conclude that the majority of A- and B2-elements are characterised by specific structural deformations and that A- and B2-elements have similar but distinct structural DNA signatures.

It is well established that ORC and ORC-Cdc6 can bend DNA (Fig. 5e)^44,45^. Given that A- and B2-elements exhibit similar DNA shapes, we hypothesised that both DNA elements should be deformed similarly when bound by ORC. Therefore, we calculated how much energy is needed to deform the A- and B2-element sequences of 331 replication origins when morphed into the ORC-DNA complex’s structure using a multivariate Ising model (Fig. S6g)^44,49,50^. The calculations consider inter-base-pair parameters (*e.g*., twist), but also intra-base-pair parameters (*e.g*., opening), an approach that was also validated for sequence-specific binding of the papillomavirus E2 DNA replication factor to DNA^50^. Our analysis revealed that, on average, the A-element sequences required 147.1 ± 0.7 kcal/mol deformation energy, while the B2-element sequences required 152.8 ± 0.9 kcal/mol and random DNAs with identical AT-content required 164.4 ± 0.4 kcal/mol. The observed differences are sufficient to impede complex formation in the non-specific DNA, and other complexes were shown to be sensitive to smaller differences^50^. Thus, our analysis indicates that both A- and B2- elements contain DNA sequences, which require low deformation energies to undergo ORC-dependent DNA bending. In summary, we show that ORC DNA binding sequences have a specific DNA shape and bendability and that these features are not only enriched in A-elements, but also in the newly identified B2-element sequences. Thus, the identified B2 sequences display many physical hallmarks of ORC binding sites.

### The DNA structure and location of B2-element sequences are associated with helicase loading efficiency

**Fig. 6:**
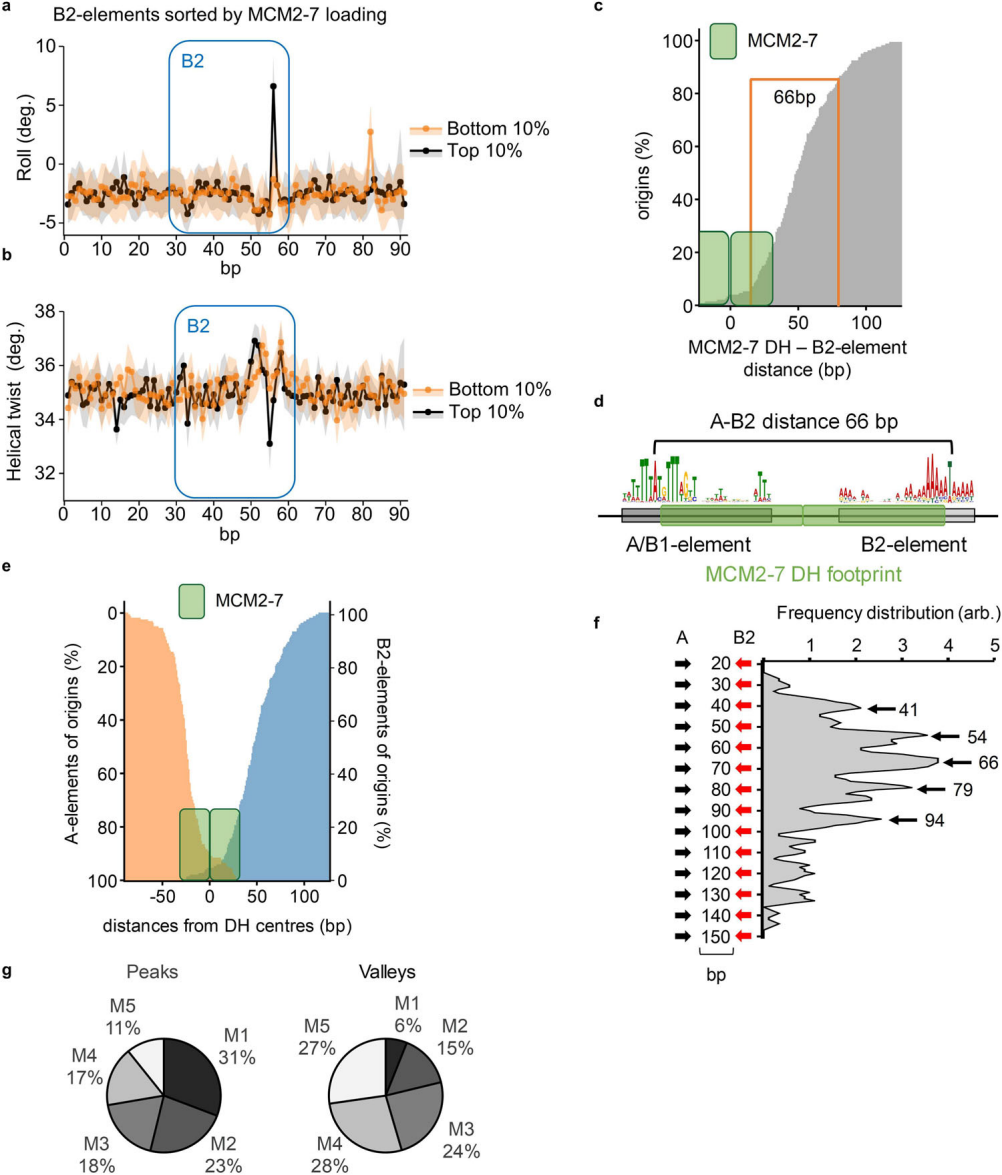
Orientation and localisation of the B2-element drive efficient origin licensing. DNA deformation at B2-elements is associated with origin licensing efficiency. Changes in roll (**a**) and helical twist (**b**) at origins with identified B2-elements (*n* = 181, each 10%) sorted by MCM2-7 loading are shown. Aligned B2-elements of origins with most (black) and least (orange) MCM2-7 ChIP-Exo 5.0 reads are depicted ± 30 bp surrounding the respective B2-element (blue box). Individual dots represent the median deformation at a given position with the surrounding 95% confidence interval shown in black or orange. **c** B2-elements are localised downstream of the MCM2-7 DH providing a window for helicase loading. A 66 bp window directly downstream of identified MCM2-7 DH centres (*n* = 164) is the main localisation for > 80% of B2-elements at origins. The footprint of the MCM2-7 DH is indicated by the green boxes. **d** Organisation of a typical *ARS* with a 66 bp distance between the A- and B2-element (counting from the conserved A/T). The MCM2-7 DH footprint is frequently located at a central position framed by the A- and B2-elements. **e** The MCM2-7DH is positioned between its loading elements. The distances of A- (orange) and (first) B2-elements (blue) from the MCM2-7 DH are shown cumulatively with the footprint of the MCM2-7 DH indicated by the green boxes. **f** A-/B2-element distances cluster in regularly spaced, distinct peaks and valleys. Peaks are indicated with black arrows and numbers indicating A-B2-element distances. **g** Highly efficient origins (highest MCM2-7 DH signals, classes M1 and M2) cluster to peak A-/B2-element distances, while less efficient origins (classes M3-M5) cluster to valleys. Pie charts highlighting the distribution of origins (M1-M5) to peaks and valleys from (**f**) based on MCM2-7 DH signal in G1-phase (licensed origins). For **a**–**c** and **e**–**g**, source data is provided as a Source Data file.

Having identified that both A- and B2-elements are characterised by strong local DNA deformations, we wanted to understand if the level of deformation at B2-elements has an influence on MCM2-7 loading. We, therefore, analysed origins that contained a correctly orientated B2-element for MCM2-7 loading and DNA deformation patterns. Origins with high MCM2-7 loading (Fig. 6a and b, top 10%, black) presented strong DNA deformations, while origins with low MCM2-7 loading (Fig. 6a and b, bottom 10%, orange) showed strongly reduced DNA deformation. Thus, the data directly link origin licensing with B2-element occurrence and DNA bendability. Repeating the same analysis for the A-elements of the same origins produced near identical plots for the tested deformation patterns (Fig. S7a and b), further highlighting the importance of a B2-element bendability for MCM2-7 DH loading in vivo.

Next, we analysed the distance between the MCM2-7 DH and correctly orientated B2-element sequences and observed that more than 80% of the B2-elements were located in a stretch of 66 bp immediately downstream of the MCM2-7 DH footprint, indicating that the identified B2-element motif can be found in a specific location (Fig. 6c). Interestingly, origin sequences with a pronounced narrowing in the minor groove width at the B2-element were enriched for high MCM2-7 loading origins (M1 and M2; Fig. S7c), while origins without B2-element were enriched for low MCM2-7 loading origins (M4 and M5; Fig. S7d), highlighting again that DNA structure is connected to function. We suggest that non-B2 origins may depend in part on MCM2-7 sliding^51^ to deliver the second hexamer, which may modulate their efficiency.

The inspection of individual origins with an A-B2 distance of ~66 bp revealed that the MCM2-7 DH is frequently located in a central position, equally or near equally spaced between the A- and B2- elements (Figs. 6d and S7e–h). A genome-wide analysis also showed that the DH is located in between the A- and B2- elements (Fig. 6e). This highlights that many budding yeast origins adopt a very compact organisation and that the location of the DH will limit ORC in accessing its primary and secondary binding sites. Thus, repetitive loading of MCM2-7 at these compact origins will be impossible as the ORC binding sites cannot be accessed.

Interestingly, when inspecting the 67 multi B2-element origins, we frequently observed a ~ 12/13 bp interval between individual B2-elements, consistent with ~1.2 helical turns in the DNA (Fig. S8a and b). For this reason, we plotted the distance between the A-element and the B2-element motif as origin frequency for all origins that contained a B2-element. This showed that most replication origins had an inter-motif distance of ~66 bp (when counting the distance between the conserved T/A of the A-/B2-elements). Surprisingly, several local maxima were observed in ~12/13 bp intervals (Fig. 6f, see region 41–91 bp), with corresponding local minima at similar intervals. Moreover, the general pattern was repeated over a distance of ~100 bp (Fig. 6f, see region 41–141 bp).

We also analysed the MCM2-7 read counts for the inter A-B2-element distances and observed a similar trend towards certain A-B2-element distances (Fig. S9a). We also found that the peaks in the A-B2 distances were enriched for high MCM2-7 loading origins (M1 and M2), while the valleys were enriched for low MCM2-7 loading origins (M3 to M5 (Fig. 6g, S9b)). Overall, not only are the individual MCM2-7 DH abundances higher for origins with peak A-B2-element distances but also 74% of the global MCM2-7 DH abundance of the tested origins is associated with peak A-B2-distances (Fig. S9c and d). Thus, the data show that the distance between the A- and the B2-element is associated with changes in the genome-wide MCM2-7 loading capability at replication origins. We suggest that the spacing will position ORC in an ideal location, which supports the recruitment of the second MCM2-7 hexamer and aligns both MCM2-7 hexamers to establish the correct DH interface (Fig. 5d and e).

We took advantage of published data to ask whether the change in DNA shape or distance between the A- and B2-elements could affect the origin function. In this study, *ARS1* (*ARS416*) was subjected to a mutational scanning approach to identify origins with improved efficiency^52^ (Fig. S10a). It was found that *ARS1-HI* displayed greater plasmid stability than *WT ARS1*, but the molecular reason for this difference was not uncovered^52^. As DNA sequence changes between *ARS1* and *ARS1-HI* were limited (Fig. S10a), we wondered whether the altered DNA sequence may affect the DNA conformation. The DNA structure analysis (minor groove width, MGW) revealed a shift from a narrow minor groove width (4.5 Å, *ARS1)* to a broader widening (5.8 Å, *ARS1-HI*) at the B2 element (Fig. S10b). We aligned the average minor groove plot to *ARS1* (Fig. S10c) and *ARS1-HI* (Fig. S10d) and overlayed these (Fig. S10e). This shows a shift of the minor groove width signature in *ARS1-HI* from 70 bp to 66 bp – effectively shortening the distance between the A- and B2-elements. Moreover, the altered DNA sequence results in greater flexibility, *i*.*e*. bending ability of the mutant DNA sequence as measured by comparative deformation analysis of the B2-elements (Fig. S10f). We suggest that a flexible DNA will better support ORC-induced bending and the 4 bp shortened A-B2-element distance shifts this origin to an inter-motif distance observed at early and efficient origins (Fig. S10e and compare Fig. 6e and f).

To test this hypothesis, we asked whether shortening the distance between the A- and B2-elements at *ARS1* would also improve the plasmid stability in vivo. Indeed, we observed improved plasmid stability in a 5 bp deletion construct (*ARS1*-5bp del) when compared to wt *ARS1*, while a B2 deletion resulted in a strong reduction (Fig. S10g). Similarly, to *ARS1-HI*, a minor groove width comparison of *ARS1*-5bp del and *ARS1* produced a shift of the minor groove width signature in the altered DNA sequence. Thus, the spacing between the A- and B2-elements and associated DNA shape changes affect origin functionality in vivo. Moreover, the data present a feasible molecular explanation of why the *ARS1-HI* mutant serves as an improved replication origin over *ARS1* in vivo (Fig. S10).

In summary, the genome-wide bioinformatic analysis has revealed a sequence motif that can predict the B2-element location at most origins with high confidence. Biophysical and structural properties are shared between the A-element and the newly identified B2 motif sequence, suggesting both functions for ORC binding. However, only in the case of B2 we identified that the B2 DNA structure is associated with changes in MCM2-7 loading (Fig. 6a and b). Moreover, the distance between primary and secondary ORC binding sites (*i.e*. A- and B2-element) is associated with differences in MCM2-7 loading efficiency and plasmid stability. Finally, the location of the B2-element is frequently adjacent to the MCM2-7 DH, suggesting that it functions in positioning the MCM2-7 DH at its origins.

## Discussion

In this study, we applied ChIP-Exo to investigate the role of ORC and MCM2-7 in DNA licensing of the entire genome during the M-G1 transition. Analysis of the genome-wide, high-resolution molecular footprints of ORC and MCM2-7 in various cell cycle stages identifies several new concepts that start to explain how faithful DNA replication can be achieved.

**Fig. 7:**
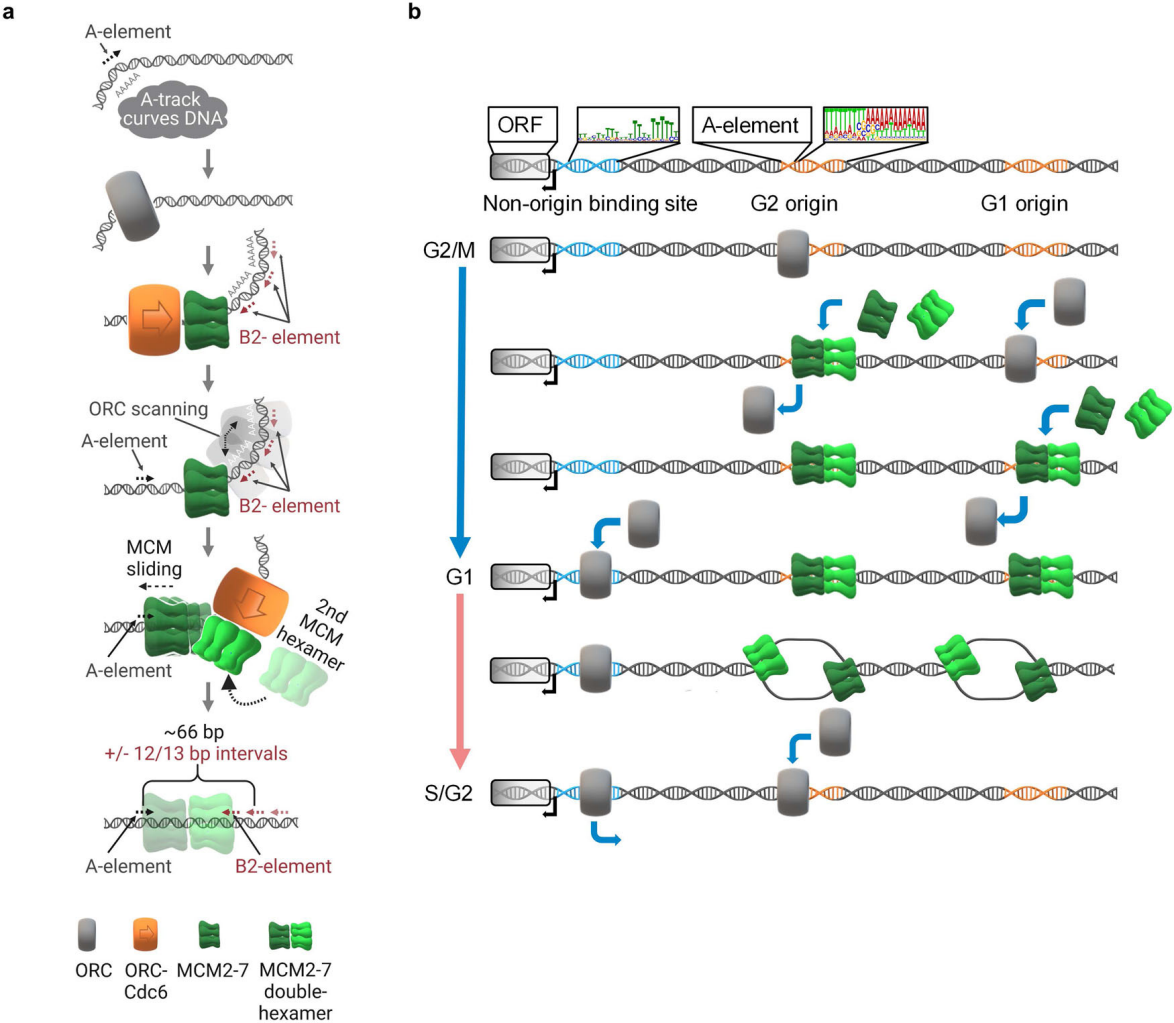
Model for origin licensing. **a** Distributive MCM2-7 helicase loading is a stepwise process. ORC recognises and binds the structurally distorted A-element of an origin to recruit the first MCM2-7 complex. Then, ORC flips over MCM2-7 to search for an adjacent B2-element with similar structural distortion properties. We propose that this ORC searching process repositions the first MCM2-7 hexamer across the A-element. Finally, ORC, bound to the B2-element, loads the second MCM2-7 hexamer and, as such, specifies the final MCM2-7 DH location. B2-elements are spread in ~1 helical turn registers from the A-element to ensure correct rotational alignment of ORC for the second MCM2-7 hexamer loading. **b** ORC is displaced from origins during MCM2-7 helicase loading to promote distributive and genome-wide origin licensing. The precise site of helicase loading is governed by the location and orientation of both the A- and B2-elements. During licensing, ORC is displaced from its location at origins. In G1-phase, the A- and B1-element sequences are covered by the MCM2-7 DH, preventing ORC rebinding and repetitive MCM2-7 loading onto the same origin. Subsequently in S-phase, the MCM2-7 DHs are activated and leave the origins as CMGs thereby freeing up the A- and B1-elements for ORC to rebind. Thus, MCM2-7-dependent displacement of ORC from its binding site leads to genome-wide redistribution of ORC and distributive MCM2-7 loading to promote balanced licensing of the genome. Once displaced from origins, ORC binds to non-origin sites, which are characterised by a T-rich motif near promoters of RNAPII-transcribed genes. **a** was produced with BioRender.com and released under a Creative Commons Attribution-NonCommercial-NoDerivs 4.0 International license.

When investigating the recruitment of ORC to origins, we found that ORC produces varying molecular footprints in different cell cycle stages. In G2-phase, ORC adopts a compact footprint, while in G1-phase, it expands, consistent with ORC-Cdc6 complex formation^23^. In G1-phase, ORC is displaced from efficient replication origins and is directed to less efficient origins and non-origin binding sites in vivo (Figs. 1 to 4 and S1–S3). We showed that ORC displacement is dependent on DNA licensing, *i.e*., the loading of an MCM2-7 DH onto the origin DNA (Figs. 2, 3, 7a and S2).

Genome-wide analyses also shed light on the number of MCM2-7 DHs at origins. We found that ~66% of all origins examined show a clear preference for a single loaded MCM2-7 DH per origin (Figs. 1, 2, and S1), while 33% of origins exhibit multi MCM2-7 binding, but their occupancy was lower than the occupancy of single peak origins, similar as recently observed^26^. Now, our work reveals that the majority of multiple MCM2-7 DH peaks ( ~ 82%) are associated with multiple ORC binding events (Fig. S1b), suggesting that ORC’s flexibility in selecting from several potential binding sites and the underlying population-based assay results in various MCM2-7 loading locations. Finally, a small number of all origins analysed ( ~ 6%) showed single ORC binding and multiple MCM2-7 DH peaks (Fig. S1c). In these cases, a process such as transcription of non-coding RNA^53^ could displace MCM2-7 from the origin and, in turn, could stimulate repetitive loading^54,55^. However, we note that multi-peak MCM2-7 origins displayed overall weak MCM2-7 loading, suggesting that repetitive loading at origins would be a rare event. Thus, we conclude that in budding yeast a single MCM2-7 DH becomes loaded per origins (Fig. 7a).

Our ChIP-Exo data and bioinformatic analysis revealed that helicase loading positions the MCM2-7 DH across the ORC binding site. In contrast, several earlier studies mapped MCM2-7 adjacent to the origin in proximity to nucleosomes^17,35^. However, since these analyses suffered from limited resolution and suboptimal signal-to-noise ratios, we suggest that ChIP-Exo approaches are more accurate due to the small footprints created in the procedures. Crucially, an immobile MCM2-7 DH at the centre of the origin is consistent with the distinct pre-RC footprint in G1-phase^24,26^, the in vitro immobility of the MCM2-7 DH under physiological salt conditions^56,57^, the requirement for high-salt buffer to observe sliding of the MCM2-7 DH^56,57^, and the structural observation that the helicase directly engages with and deforms the DNA^28^.

So far, B2-elements have been poorly defined with respect to function, location, and composition. Our in-depth bioinformatics analysis identified a significantly improved B2-element sequence and suggests that ORC not only recognises its secondary ORC binding site based on the DNA sequence but also based on DNA shape and bendability. Indeed, origins that load MCM2-7 less efficiently have a reduction in DNA structure in their B2 motif (Fig. 6a and b). When comparing our B2 motif to previously identified motifs^33^ (Fig. S5), it is clear that the new motif allows a much more robust identification of B2 sequences. Finally, the motif sequence and location are consistent with previous mutational analysis of the B2-element^7,52^. Why did we not identify a B2 site at all origins? Recently, it was found that two distal A-elements can load single-MCM2-7 hexamers, which consequentially translocate and form a double-hexamer^51^. As such, we suggest that a subset of B2-less origins may bring MCM2-7 hexamers in proximity via MCM2-7 translocation.

The identification of the B2 motif in combination with the ChIP-Exo data also provides insights into the mechanisms that position MCM2-7 DHs at origins (Fig. 7a). Based on the identified ORC footprint and consistent with a cryo-EM structure of the OCCM complex^42^, we suggest that the first MCM2-7 hexamer becomes loaded adjacent to the A-element (Figs. 2, 6d, e and 7a). In turn, the A-element serves as a border of the MCM2-7 loading site. It has been shown that ORC is released from its primary binding site and, supported by an ORC-MCM2-7 interaction, flips to the other side of the origin (*e.g*., from the C-terminal site of MCM2-7 to the N-terminal site of MCM2-7)^8,15^. This flip allows ORC to scan the DNA for its secondary binding site - the B2-element^58,59^. Thus, we conclude that ORC’s movement repositions the loaded MCM2-7 hexamer across the primary ORC binding site, which blocks repetitive MCM2-7 loading at this origin (Fig. 7a). Since the identified B2 sequence has a specific DNA shape that is associated with improved helicase loading, has similarities with an inverted A-element (Fig. 5a and b), and is located proximal to the MCM2-7 double-hexamer (Fig. 6e), we suggest that B2-elements functions as the loading site of the second MCM2-7 hexamer and that the MCM2-7 DH remains in a static position post-loading (Fig. 7a). Considering that the MCM2-7 DH overlaps more with the A-element than the B2-element and, in this way, renders the A-element inaccessible for ORC, we suggest that the B2-element is the most important determinant for positioning of the helicase at the origin.

With the identification of the B2 motif sequence, we could explore how the specific arrangement of the A- and B2- elements correlate with MCM2-7 loading. We found that specific distances between the elements are associated with enhanced MCM2-7 loading, and in turn, a reduction in spacing was associated with less DNA licensing (Figs. 6, S7 to S10). Most replication origins had an A-B2-element distance of 66 bp, and 80% of the origins presented an A-B2-distance between 41–91 bp (Figs. 6 and S6). We postulate that an optimal A-B2 spacing results in the correct rotational alignment of both MCM2-7 hexamers. This, in turn, supports the establishment of the stable DH interface, while a suboptimal distance could result in hexamer misalignment and non-functional helicase complexes. Although the distance between the A- and B2-element vary (41–91 bp), both sites are found overall in close proximity (Figs. 6 and S7–S9), highlighting that in vivo budding yeast replication origins are very compact at ~80 bp in length (including the complete A- and B2-element sequences). In comparison, bacterial replication origins have a length of 200-1000 bp^60,61^. In the much more relaxed in vitro setting, which omits nucleosomes and other DNA binding proteins, an A-B2 distance of 133 bp was found to be optimal for MCM2-7 loading^34^. Therefore, our data suggest that the local chromatin environment (*e.g*., nucleosomes) restricts MCM2-7 loading to a surprisingly small stretch of DNA in vivo^62^.

Furthermore, MCM2-7 loading could be regulated by the number of B2-elements and their sequence composition. We suggest that multiple secondary ORC binding sites (up to 5) may support the ORC flip process due to the multiplicity of binding sites, while the gradient in B2-element sequence conservation we observed may support ORC’s search for the correct binding site (Fig. S8). In support of the first concept, a synthetic *ARS1* replication origin with multiple B2-elements was more efficient in DNA replication than the wt *ARS1*^52^.

Our data provide significant insight into the regulation of DNA licensing of chromosomes. We discovered that MCM2-7 is loaded across > 90% of primary (A-element) (Figs. 2, S7, and S8) and > 68% of secondary (B2-element) ORC binding sites (Fig. 6d and e). Consistently, we identified that ORC becomes displaced from high-efficiency origins (Fig. 7b). Importantly, this displacement was only observed under permissive DNA licensing conditions, demonstrating that MCM2-7 loading leads to ORC removal (Figs. 2 and 3). Interestingly, we observed that displaced ORC binds to non-origin binding sites, which could serve as repository sites or support non-licensing roles of ORC (Fig. 7b)^63,64^. Our data show that in G1-phase, C3/C4 origins display increased ORC binding. However, this could be for multiple reasons, *i*.*e*. chromatin changes, redistribution from C1/C2 origins or increased ORC expression. Consistently with the ORC displacement model, we observed a single MCM2-7 peak at most replication origins, blocking repetitive MCM2-7 loading at origins.

The ORC displacement data (Figs. 2 and 3) are consistent with 1.) early ChIP experiments that showed a reduction of ORC at origins during the G2/M–G1-phase transition, but were back then interpreted as epitope exclusion due to the use of ORC antibodies^65^; 2.) the identification of G1-only ORC footprints^17^; 3.) in vitro experiments that indicated helicase loading-dependent ORC release from origins^8,34^; and 4.) the observation that ORC and Cdc6 interact dynamically with chromatin, while the MCM2-7 DH is more static^66^. Thus, our novel mechanistic insight and previous observations are consistent with the ORC displacement concept, bridging in vitro and in vivo observations systematically for the first time.

In summary, our data reveal that DNA licensing leads to individual MCM2-7 DH loading events and uncover the underlying mechanisms - MCM2-7 being placed above the ORC binding site. As such, our work identifies that DNA licensing follows a distributive model–limiting the maximal licensing of each origin. A distributive licensing model is also appealing to organisms, such as humans, as it avoids over-licensing of a few high-efficiency origins and promotes a more even distribution of MCM2-7 DHs.

## Methods

### Yeast strains and plasmids

Yeast strains, plasmids, and DNA sequences used in this study are listed in the supplementary information. *Saccharomyces cerevisiae* strains (W303) were used throughout the manuscript and grown at 30 °C in full medium (YPD) if not stated otherwise. All genomically tagged strains were integrated into their endogenous locus and confirmed by colony PCR and Western Blot if applicable.

### Plasmid mutagenesis

The QuikChange II Site-Directed Mutagenesis Kit (Agilent) was used to generate the *ARS1* deletion mutant. Clones were screened by restriction digest using StuI (NEB) and validated by whole plasmid sequencing.

### Plasmid propagation assay

*ARS1*-containing *CEN4* plasmids were transformed into YC510 cells and cultures were grown to saturation overnight in SDC-URA media^67^. Cultures were diluted, cells counted in a Neubauer chamber, and two 5 ml cultures of YPD (14 ml round-bottom tubes, BD) were inoculated with 1×10^5^ cells/ml. The following steps were performed in technical duplicates in addition to biological replicates: 10 μl from each culture was diluted 1:10 in PBS and plated to achieve ~400 cells per plate on selective (SDC-Ura) and ~200 cells per plate onto non-selective media (YPD). The remaining cultures were grown overnight (16–24 hrs, 30 °C, 220 rpm, usually about 11 generations). Cultures were placed on ice and counted to calculate the number of generations and strain doubling time. Counted cells were again inoculated in two 5 ml cultures of YPD with 1 × 10^5^ cells/ml and plates seeded as above. This process was repeated to generate 48 and 72 hrs time points (~33 generations). Colonies on plates were counted after three days at 30 °C and plasmid propagation was calculated as the relative proportion of cells harbouring the URA-containing plasmids over all cells grown in non-selective media.

### ChIP and ChIP-Seq experiments

All ChIP-qPCR experiments were performed as follows^68^. *S. cerevisiae* strains were grown in appropriate media and arrested in G1-phase by two hours treatment with α-factor at 25 °C, 220 rpm. Cells were spun down (500 g, 2 min, RT), washed once with pre-warmed media, and released in 100 ml warm media (25 °C). For arrest in G2/M-phase, cells were treated with nocodazole (final conc. 15 ug/ml) for two hours at 25 °C, 220 rpm before cross-linking with 1% formaldehyde (Sigma) for 15 min at RT with mild agitation. Cross-linking was stopped with glycine (0.25 M final conc.) and incubated for 5 min at RT. Cells were harvested (2500 g, 2 min, RT), washed with 1x TBS, and flash-frozen in liquid nitrogen until use. Pellets were thawed on ice, resuspended in 0.8 mL of FA-Lysis buffer (low salt, 50 mM HEPES-KOH pH 7.5, 150 mM NaCl, 2 mM EDTA pH 8.0, 1% Triton X-100 (v/v), 0.1% Na-Deoxycholate (w/v), and 0.1% sodium dodecyl sulphate (w/v)), supplemented with a protease inhibitor cocktail, and mixed with equal amounts of glass beads. Cell lysis was done on a MP FastPrep-24 5 G with 3 × 45 sec cycles (7 m/s) and 2 min breaks on ice. Lysates were sonicated using a Bioruptor (Diagenode) 3 × 15  min (30 sec on/off) with 5 min breaks on ice to yield chromatin size between 0.25–0.5 kbp. The lysate was cleared by centrifugation twice (5 min and 10 min, 14,500 g at 4 °C). After measuring and equalising protein amounts, samples were incubated with a specific antibody against the protein/tag of interest (anti-FLAG 1/300, Sigma, F-1804) for 1.5 h on a roller at room temperature (25 °C). Subsequently, 15 μL pre-equilibrated ProteinG Dynabeads (Thermo) were added and the samples were incubated for 1 h at 25 °C. Beads were collected on a magnetic rack and washed with buffer (2x FA-Lysis low salt, 2x FA-Lysis high salt [50 mM HEPES-KOH pH 7.5, 500 mM NaCl, 2 mM EDTA pH 8.0, 1% Triton X-100 (v/v), 0.1% sodium deoxycholate (w/v), and 0.1% sodium dodecyl sulphate (w/v)], 2x TLEND [10 mM Tris-HCl pH 8.0, 250 mM LiCl, 1 mM EDTA pH 8.0, 0.5% NP-40 (v/v), and 0.5% sodium deoxycholate (w/v)]) and 1x TE. Next, DNA was eluted from beads using ChIP elution buffer (50 mM Tris-HCl pH 7.5, 10 mM EDTA pH 8.0, and 1% sodium dodecyl sulphate (w/v)) and vigorous shaking at 65 °C for 20 min. After the addition of Proteinase K, samples were incubated for 2 h at 37 °C, followed by 12−16 hrs of incubation at 65 °C. Samples were purified using the QIAquick PCR Purification kit (Qiagen) according to the manufacturer’s instructions and diluted 1/5 to 1/10 before qPCR on selected origins (Biorad CFX96). If samples were used for ChIP-sequencing instead, standard library generation was performed from the purified eluates using the NEBNext® Ultra™ II DNA Library Prep Kit for Illumina following the manufacturer’s instructions. Libraries were checked on a 2100 Bioanalyzer (Agilent) before pooling and Illumina next-generation sequencing on a HiSeq 2500 sequencer (Illumina).

For time course experiments using the *cdc20* mutant, cells were grown in minimal media lacking tryptophan and methionine (SDC-Trp-Met) at 30 °C to OD_600_ 0.5. Cells were then transferred into YPD to induce a G2-phase arrest for 2.5 h at 25 °C. During the last 20 min of arrest, the media was supplemented with α-factor. After washing, cells were released into fresh SDC-Trp-Met complemented with α-factor and samples for ChIP-qPCR were taken at 15 min increments and processed as described above.

For time course experiments using the *cdc6-1 and cdc46-1 ts-* mutant, cells were grown in YPD at 23 °C (permissive temperature) to OD_600_ 0.5. Cells were then transferred to the non-permissive temperature (37 °C) for 3 hrs^31^. After one hour, media was supplemented with α-factor (5 ug/ml final conc.) or nocodazole (15 ug/ml final conc.) to induce the cell cycle-specific arrest. Samples for ChIP-qPCR and ChIP-seq were taken and processed as described above.

### ChIP-Exo 5.0 experiments

Cells were grown in 100 ml YPD from diluted overnight cultures to OD_600_ 0.6 at 220 rpm and 30 °C followed by two hours α-factor arrest (final conc.: 5 ug/ml) at 25 °C, 220 rpm. Cells were spun down (500 g, 2 min, RT) washed once with pre-warmed media and released in 100 ml warm media (25 °C) and time points were taken as indicated. For arrest in G2/M-phase, cells were treated with nocodazole (final conc. 15ug/ml) for two hours at 25 °C, 220 rpm. Cells were crosslinked, washed, lysed, and sonicated as described for ChIP. After immunoprecipitation and washing as described above, samples on beads were resuspended in 10 mM Tris-HCl (pH 8.0), transferred into 0.2 ml PCR strips, and the remaining steps were performed using a ThermoMixer C (Eppendorf) with a 96-well block as follows (all regents were purchased from NEB if not stated otherwise)^18^: A-tailing (each reaction in 50 μl; 15 U Klenow fragment (-exo), 5 μl NEB buffer 2, and 100 μM dATP) was performed for 30 min at 37 °C, 1050 rpm. Beads were collected on a magnetic stand, the supernatant discarded, and the beads washed with 150 μl cold 10 mM Tris-HCl (pH 8.0). First adaptor ligation (each reaction 45 μl; 1200 U T4 DNA ligase, 10 U T4 PNK, 4.5 μl 10x NEBNext Quick ligation reaction buffer, and 375 nM adaptor mix (ExA2_iNN / ExA2B)) was incubated for 1 h at 25 °C, 1050 rpm. Beads were collected on a magnetic stand, the supernatant discarded, and the beads washed with 150 μl cold 10 mM Tris-HCl (pH 8.0). Subsequently, a fill-in reaction was set up (each reaction 40 μl; 10 U phi29 DNA polymerase, 4 μl 10x phi29 reaction buffer, 0.2 mg/ml BSA, and 180 μM dNTPs) mixed with the beads and incubated for 20 min at 30 °C, 1050 rpm. Beads were collected on a magnetic stand, the supernatant discarded, and the beads washed with 150 μl cold 10 mM Tris-HCl (pH 8.0). After that, DNA on beads was digested using λ exonuclease (each reaction 50 μl; 10 U λ exonuclease, 5 μl 10x λ exonuclease reaction buffer, 5 μl Triton X-100 (1% v/v), and 2.5 μl DMSO (5% v/v)) for 30 min at 37 °C, 1050 rpm. Beads were collected on a magnetic stand, the supernatant discarded, and the beads washed with 150 μl cold 10 mM Tris-HCl (pH 8.0). DNA was eluted from beads (each reaction in 40 μl; 3 μl ProteinaseK ( > 10 mg/ml, Sigma), 25 mM Tris-HCl (pH7 .5), 2 mM EDTA (pH 8.0), 200 mM NaCl, and 0.5% sodium dodecyl sulphate (w/v)) for 25 min, 65 °C, 1200 rpm, and decrosslinked overnight (15.5 hrs) in a PCR cycler at 65 °C. To prepare for second adaptor ligation, DNA was cleaned-up using AMPure magnetic beads (Beckman Coulter). Second adaptor ligation was set up in 20 μl reactions (1200 U T4 DNA ligase, 2 μl 10x T4 DNA ligase buffer, 375 nM adaptor mix (ExA1-58/ ExA1SSL-N5)) and incubated for 1 h at 25 °C, 1050 rpm before clean up with AMPure beads. Finally, libraries were amplified in 25 μl reactions (2U Phusion Hot Start polymerase (Thermo Scientific), 5 μl 5x Phusion HF buffer, 200 μM dNTPs, and 500 nM of primers (P1.3 and P2.1)) mixed with 15 μl purified DNA. PCR conditions were as follows: 20 s, 98 °C initial denaturation and 18 cycles of 20 s, 98 °C, 60 s, 52 °C, and 60 s, 72 °C.

After amplification, DNA libraries were gel extracted (2% agarose gel, DNA with a molecular weight between 200–500 bp) using the NEB Monarch PCR and DNA Cleanup Kit according to the manufacturer’s instructions to allow elution with smaller volumes. Libraries were quantified using Qubit fluorometry (Thermo Fisher, dsDNA HS Assay) and qPCR (KAPA Illumina library quantification) before pooling and Illumina next-generation sequencing (HiSeq 2500). Bam/ bai files were visualised using the Integrative Genomics viewer^69^.

### Bioinformatical Analysis

#### ChIP-Exo and ChIP-sequencing

The raw sequencing reads were assessed for quality using FastQC (ver. 0.11.5)^70^. ChIP-Exo-specific quality controls were performed using ChIPexoQual^71^. Alignment of reads was performed with Bowtie2 aligner (ver. 2.3.5) using default settings^72^. Bigwig files were generated for visualising with genome browser using deepTools (ver. 3.2.1)^73^.

Read distributions of ChIP-Exo data were visualised using CHASE using standard parameters^74^. Sequence logos and frequency plots were prepared with WebLogo using standard parameters^75^. De-novo motif finding, motif enrichment, and motif scanning were done using MEME Suite (ver. 5.3.0))^76^. ChIP-Exo-derived bed files contained binding coordinates of ORC and MCM2-7 DHs. Footprints and binding heat maps were produced with ChExMix (ver. 0.45) using standard parameters and --kldivergencethres −3 or −10, and --nomotifs^77,78^.

Origin and non-origin distribution plots were produced by analysing ORC peaks from origins and non-origins using ChIPpeakAnno Bioconductor package^79^. For quantification of ORC binding in classes C1-C4 (quartiles) and MCM2-7 binding in classes C1-C4 (quartiles) and M1-M5 (quintiles) per base read depths were analysed in 100 bp windows surrounding the centre of protein binding. Signal quantification of MCM2-7 binding to origins was achieved by analysing normalised base read depths in a 60 bp vs 800 bp window surrounding the centre of protein binding^26^. For calculations involving replication timing, replication times as previously published^36,80^ were extracted for A-element sequences of the tested origins.

All distance calculations involving A- and B2- elements were centred on the central adenine of the ACS motif and the central T of the B2-element motif and only the distance between the conserved T/A of the A-/B2-elements was counted. Distances were summarised as median, 1st, and 3rd quartiles with error bars as detailed in the figure legends. For the analysis in Figs. 6d, S9a, and b, plots were smoothed with a sliding window (width 2.5) for better visibility without changing the location of per bp values.

Origin coordinates were extracted from OriDB^3,4^. A-element positions were correlated with our ChIP-Exo data and consequently corrected based on individual ORC and MCM2-7 footprints where necessary. Origins with no MCM2-7 occupancy were excluded.

#### B2-element identification

To discover B2-elements in a genome-wide manner, we first manually collated well-known *ARS* sequences to be able to extract an initial B2-element. This B2-element was then used together with positional data of the MCM2-7 DHs from ChIP-Exo datasets for de novo motif discovery (MEME)^81^ and validation (scanning, FIMO)^82^ in an iterative process until B2-elements were predicted in a robust and specific manner judged by sequence composition, location, and performance against previously identified motifs. E-values and p-values (high specificity and minimal false discovery rate (random motif discovery)) and alignments to the SacCer3 genome were used to assess prediction quality.

#### DNA property analyses

To test for changes in DNA properties (minor groove width, helical twist, propeller twist, and roll) along desired sequences (A- and B2-elements), data was extracted from GBshape^48^ ± 30 bp surrounding the motifs. To calculate local electrostatic potential changes, data was extracted from DNAphi^47^ ± 30 bp surrounding the motifs. To calculate local sequence conservation amongst *Saccharomyces* strains, data was extracted from PhastWeb^43^. Then, signals were calculated and plotted per base pair (median) along with the surrounding 95% confidence interval.

For the analyses of *ARS1* and *ARS1-HI* in Fig. S10, minor groove widths were predicted by DNAphi^47^. Then, the minor groove plots were smoothed with a sliding window (width 2) for better visibility without changing the location of per bp values and consensus plots for the A- and B2-elements were overlaid.

#### DNA deformation analysis

Deformation energies for A- and B2- elements, 1000 randomly generated control sequences (same GC-content), as well as for the B2-elements of *ARS1* and *ARS1-HI* (sequences below) were calculated based on the ORC-72bp cryo-EM model (PDB: 5ZR1)^44^. Sequences were analysed with CURVES + ^49^ followed by conformational flexibility calculations using a multivariate Ising model^50^ that incorporates relevant local effects (bimodality and nearest-neighbour coupling). Note that this model not only includes inter-base-pair parameters (*e.g*., twist), but also intra-base-pair parameters (*e.g*., opening). The energies have been computed by running the Ising model along the entire 41-bp DNA sequence present in the ORC-DNA cryo-EM model (PDB: 5ZR1)^44^. Note that energy terms of the multivariate Ising model cannot be fully projected onto one base-pair step, but the plotted terms also account for coupling interactions with neighbouring base-pairs.

### Statistical Analysis

All ChIP-qPCR experiments presented are derived from at least three biological replicates. Values represent the average of the replicates and error bars are calculated as standard deviation, if not mentioned otherwise. ChIP-Exo experiments are derived from two biological replicates. For quantification of ORC and MCM2-7 DH occupancies, statistical tests are detailed where appropriate. All other statistical analyses are detailed in the Material and Methods section and figure legends.

### Reporting summary

Further information on research design is available in the Nature Portfolio Reporting Summary linked to this article.

### Supplementary information

Supplementary Information

Peer Review File

Description of Additional Supplementary Files

Supplementary Data 1

Reporting Summary

### Source data

Source Data

## Data Availability

The raw sequencing data generated in this study have been deposited in the GEO repository database under accession code GSE240779. Coordinate files for MCM2-7-DH, ORC, A-elements and B2-elements are available (Supplementary Data 1). Source data are provided in this paper.

## Code availability

The code for the Ising model computations can be found here (https://github.com/XiPacha/Ising-model).
