# Supplementary material for: MCM2-7 loading-dependent ORC release ensures genome-wide origin licensing
Source: Nat Commun. 2024 Aug 24;15:7306. doi: 10.1038/s41467-024-51538-9 (PMC11344781; doi:10.1038/s41467-024-51538-9)
Supplement: Supplementary file 1 — Supplementary Information [file 41467_2024_51538_MOESM1_ESM.pdf]

SUPPLEMENTARY INFORMATION

MCM2-7 loading-dependent ORC release ensures genome-wide origin licensing

L. Maximilian Reuter[1,a*], Sanjay P. Khadayate[2], Audrey Mossler[1], Korbinian Liebl[2], Sarah V. Faull[1], Mohammad M. Karimi[3,4] and Christian Speck[1,4*]

Content:

Supplementary Discussion

Supplementary Figures and Figure Legends (1-10)

Supplementary Tables (1-4)

Supplementary References

SUPPLEMENTARY DISCUSSION

Alternative ORC recruitment modes

While we identify an improved B2-element containing sequence and structural features consistent with ORC binding, we do not exclude the possibility that ORC could be recruited in a B2-independent way to origins. Indeed, it is known that ORC can become recruited by other mechanisms, *e.g.*, through ORC chromatin modifications[1] or ORC transcription factor interactions[2]. In addition, much more distant B2 motifs could be accessed through extensive sliding of helicase complexes or DNA coiling[3,4].

Non-origin binding site recognition of ORC

Noteworthy, DNA licensing-dependent ORC displacement (Figure 2, 3, and S2) and increased transcription of *ORC* genes in G1-phase, generate an ORC pool that can interact with non-origin sites, such as transcription-start sites in promoters that are free of nucleosomes[5,6] (Figure 4F and S3), the same location where human origins are positioned[7]. Here, ORC could potentially influence gene regulation during G1- and S-phase of the cell cycle. Indeed, we found that ORC binds to the *MAT* locus specifically in G1-phase (Figure S3A, S3C and S3D), an event that is a key step in establishing local silencing[5]. Curiously, ORC binding to protein-coding regions of highly transcribed metabolic genes has been detected in cycling cells[8], indicative of additional non-origin ORC binding sites with potential further functions.

ORC must recognise non-origin binding sites in a different way than origin binding sites, as their binding motifs are very different (compare Figure 2D with 4C). In this context, it has been observed that removal of the 19-amino acid insertion helix in the Orc4 subunit reduces the DNA binding specificity of budding yeast ORC to A-elements dramatically[9,10]. The mutant ORC prefers a T-rich sequence and shows enrichment at transcriptional start sites, which are both similar to the non-origin binding sites identified here. As such, it appears possible that the ORC-non-origin binding site interactions are less dependent on the insertion helix of Orc4, which is absent in both human ORC and the humanised version of yeast ORC[9].

Our data show that in G2-phase ORC associates only with a subset of origins, which are enriched for the most conserved A- and B1- element sequences, while origins containing less conserved ORC binding sites were bound with lower probability (Figure 2 and S2). This indicates that the cellular pool of ORC is not sufficient to cover all ORC binding sites with equal probability. Although the number of ORC molecules exceeds the number of chromosomal replication origins, every replication origin is exposed to >600-fold excess of non-origin DNA, which will reduce the pool of ORC that can bind to the origin DNA. Indeed, ORC has limited

sequence specificity, as it was observed that ORC-dependent MCM2-7 loading can occur at non-origin sequences[11]. Thus, ORC concentration, its sequence specificity, the A-element quality, and chromatin access[12] will regulate ORC's ability to bind a replication origin. This suggests that *in vivo* ORC is a limiting factor, meaning that origins compete for ORC binding. Consistently we did not observe binding to non-origin binding sites when DNA licensing was blocked (Figure 4E).

How could a limiting ORC concentration regulate DNA licensing *in vivo*? Our study found that cluster C1 origins with very good A-element sequence conservation and efficient helicase loading (Figure 2) also replicate early in S-phase (Figure S2). Temporal order in DNA licensing is well placed to facilitate genome stability, as it would ensure that specific origins become licensed early or with higher efficiency. Indeed, we observed that cluster C1 origins are enriched for telomeric replication origins, which rely on efficient DNA licensing to ensure genome stability (Figure S2B). Moreover, changing the sequence specificity of yeast ORC also affects replication timing[13]. Thus, one can speculate that the specificity of the ORC-DNA interaction influences DNA licensing and may also affect the time of origin firing, which has been suggested to appear in human cells[14].

LIMITATIONS OF THIS STUDY

The identification and localisation of structural elements that drive DNA licensing in our study are limited by the correct annotation of the A- and B2-elements within the replication origin[15]. Although we improved the prediction of the B2-element (Figure S5)[16], the limited sequence specificity of ORC can result in the mis-annotation of A- and B2-elements. We found that some OriDB-annotated A-elements were outside ORC peaks. In addition, the presence of multiple B2-elements combined with a non-exhaustive detection of all potential B2-elements by our motif means that the average A-DH-B2-element distance found might not predict every origin correctly. Although we observed very sharp peaks for the majority of origins, arguing towards a highly specific helicase loading and immobile MCM2-7 DH in a population of cells, we sometimes found multiple ORC and MCM2-7 peaks at low efficiency origins, limiting the exact prediction of DNA elements at late and dormant origins. Thus, since population-based genomic studies rely on averaging binding events, the data may not fully reflect the situation in individual cells.

SUPPLEMENTARY FIGURES AND FIGURE LEGENDS

Reuter et al Figure S1

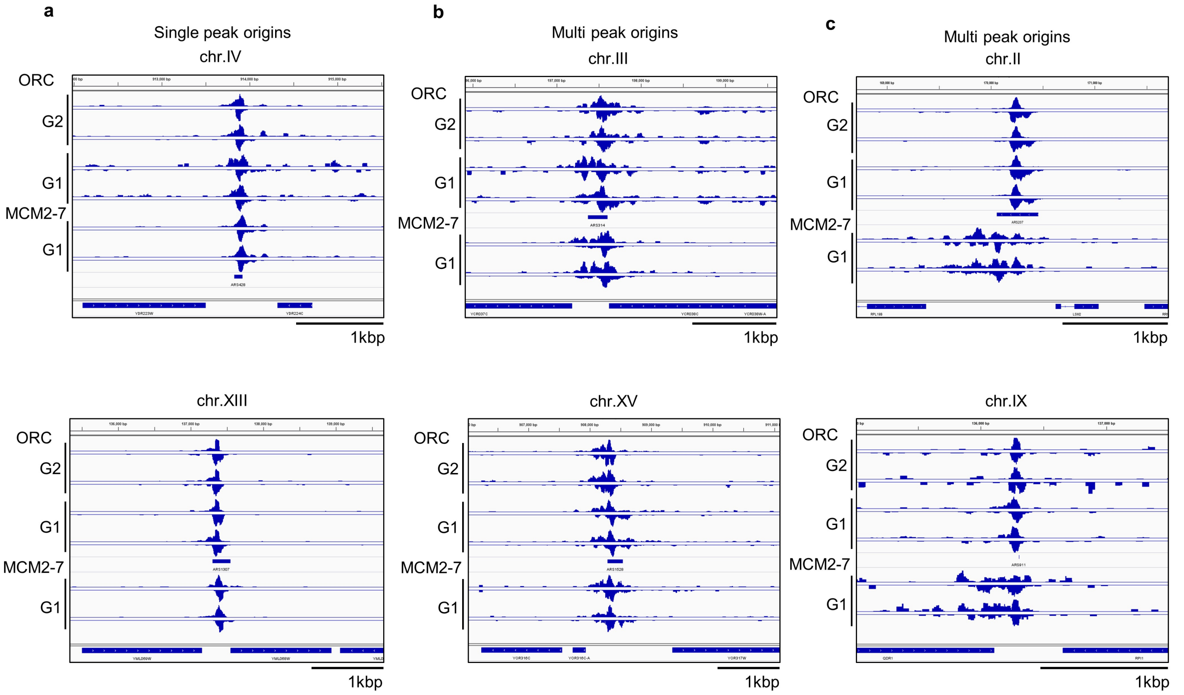

Figure S1: Concerted binding of ORC and MCM2-7 at origins.

ChIP-Exo 5.0 single traces of ORC (Orc2, G2-, and G1-phase) and MCM2-7 DH (Mcm4, G1-phase) binding to origins. Examples of (A) single-peak and (B) multi-peak origins are shown. (C) Examples of single and multiple ORC peaks at multiple MCM2-7 DH peak-containing origins are shown. Traces were visualised by IGViewer (ver. 2.4.14) with 1 kbp scale bars for reference. Source data is provided as a Source Data file.

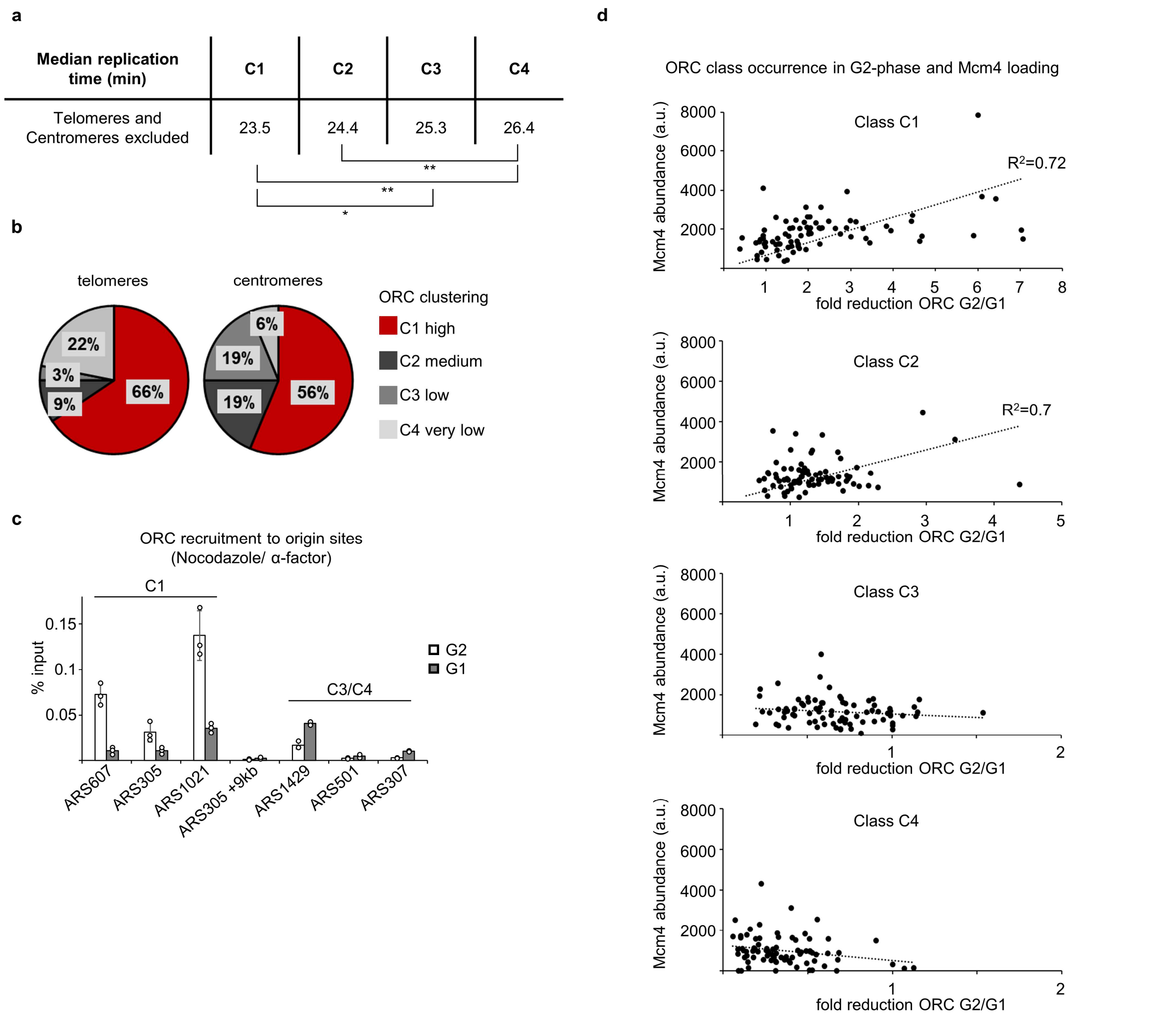

Figure S2: Loss of ORC recruitment in G1-phase correlates with DNA licensing and replication time.

(A) Early origin firing correlates with efficient ORC binding in G2-phase. The median replication times for ChIP-Exo-derived ORC binding clusters C1-C4 origins were calculated from[17]. Significance levels were calculated using two-sided Mann-Whitney U tests from 284 origins (from left to right n= 53, 79. 80, 72; *: $p < 0.05$, **: $p < 0.01$). (B) ChIP-Exo-derived ORC binding clusters C1-C4 were analysed for their occurrence at centromeres and telomeres (closest origin). (C) ORC is displaced from early origins in G1-phase. ChIP-qPCR analysis of Orc2 on selected early (*ARS1021*, *ARS607*, and *ARS305*, cluster C1), late origins (*ARS1429* and *ARS307*, cluster C4; *ARS501*, cluster C3), and a control region (*ARS305+9 kbp*) in G2/M-phase (nocodazole) and G1-phase (α-factor) arrested cells. Presented are the average and standard deviation of biological replicates (n=3). (D) Reduction of ORC binding in G1-phase correlates with MCM2-7 DH abundance at target origins. Plotting the ChIP-Exo-derived MCM2-7 DH abundance (a.u.) against ORC reduction G2-/G1-phase, ORC binding classes C1 and C2 show a correlation between MCM2-7 DH binding and ORC displacement. Classes C3 and

C4 show an anti-correlation, with ORC binding increased in G1-phase, to support licensing of G1 origins. Regression lines are indicated (black) with the regression coefficient where appropriate. Source data are provided in the Source Data file.

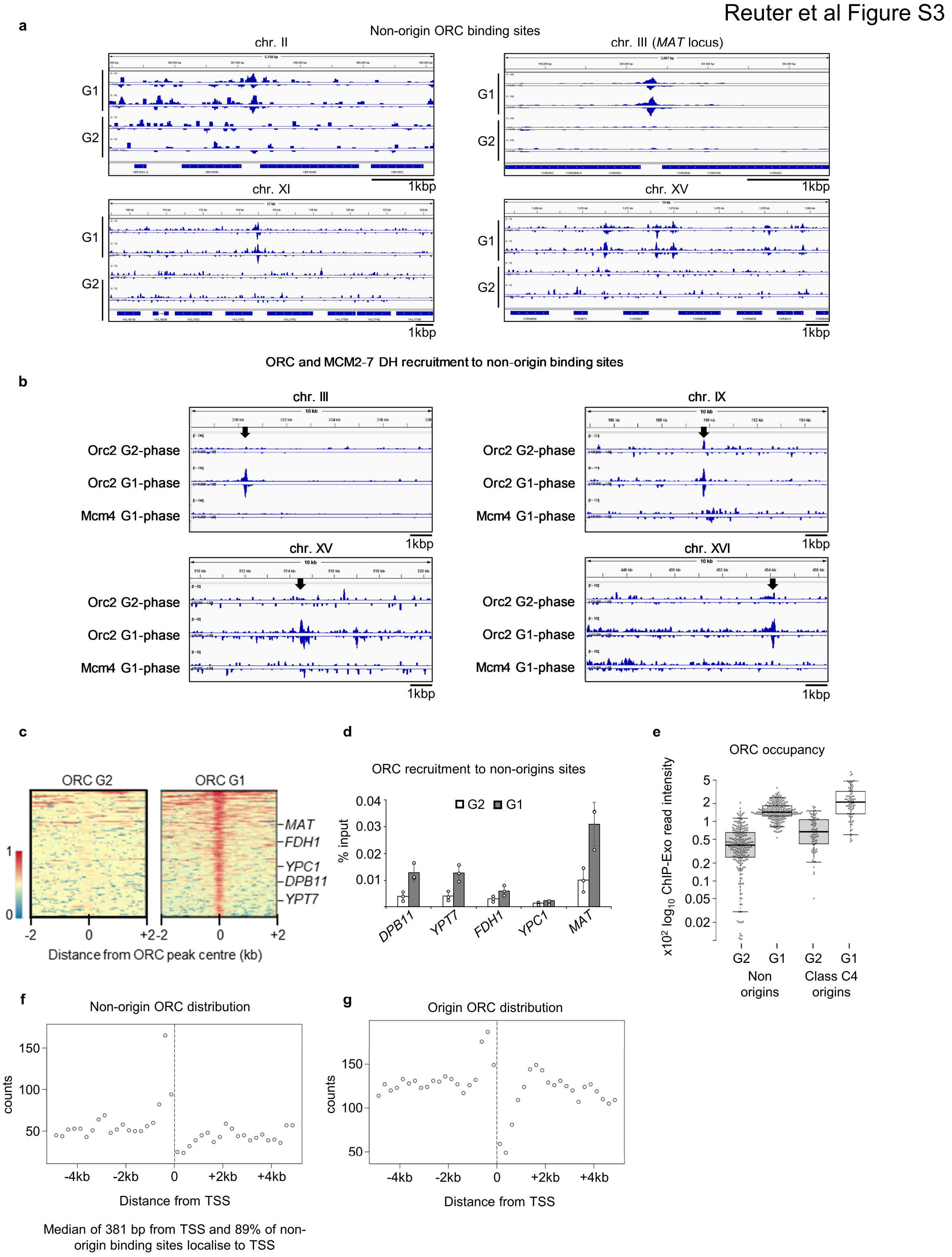

Figure S3: ORC binds to non-origin binding sites.

(A) ChIP-Exo 5.0 single traces of ORC binding (G2- and G1-phase) to non-origin binding sites. Examples were visualised by IGViewer (ver. 2.4.14) with 1 kbp scale bars for reference. (B) ChIP-Exo 5.0 single traces of ORC (G2- and G1-phase) and MCM2-7 DH binding (G1-phase)

to non-origin binding sites. Examples were visualised by IGViewer (ver. 2.4.14) with 1 kbp scale bars for reference. (C) ChIP-Exo 5.0-derived heat maps of Orc2 binding in G1- and G2-phase to 242 non-origin sites as in Figure 4A, highlighting individual sites used in this study. (D) G2/M- (nocodazole) and G1-phase (α-factor) arrested cells showcase the specific ORC binding to selected non-origin sites in G1-phase by ChIP-qPCR. Presented are the average and standard deviation of biological replicates (n=3). (E) ChIP-Exo 5.0-derived, genome-wide quantification of ORC binding to non-origin binding sites and cluster C4 origins in G2/M- and G1-phase. Individual values, as well as the median, 1st, and 3rd quartiles, are shown. Whiskers extend to 5th and 95th percentile. (F) ORC is recruited to promoters at non-origin binding sites. ORC peak distribution at non-origin binding sites was plotted around transcription start sites (TSS) ±5 kbp. (G) ORC peak distribution at origin binding sites was plotted around transcription start sites (TSS) ±5 kbp. For (a-b) and (d-g), source data is provided as a Source Data file.

Reuter et al Figure S4

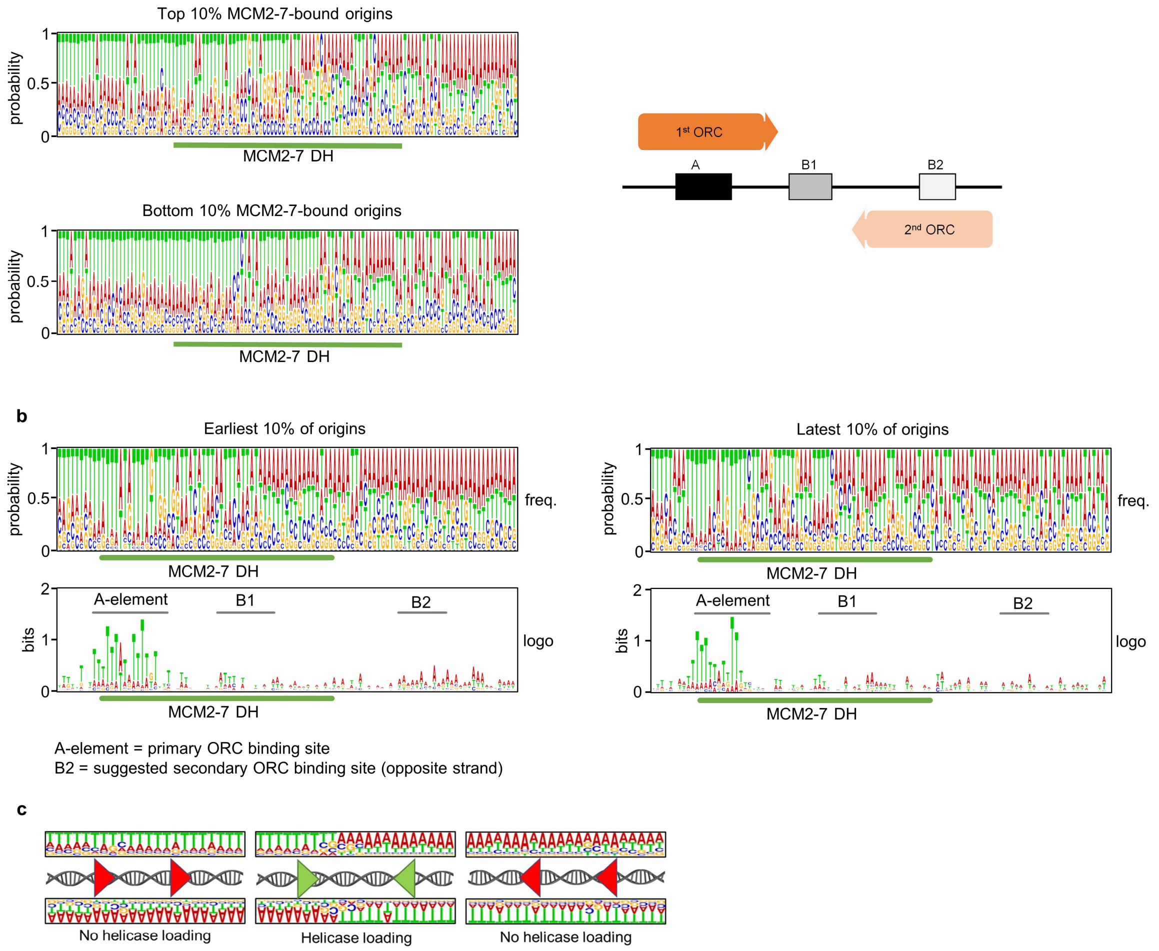

Figure S4: High MCM2-7 DH abundance and early–activated origins exhibit higher similarity to the standard A-element.

(A) Nucleotide frequency plots of the top and bottom 10% of MCM2-7 DH binding events (aligned by MCM2-7 DH centre and area covered by MCM2-7 DH in green) with nucleotides colour-coded (T in green, A in red, G in yellow, and C in blue). A model with the canonical (orange) and the potential second (pale orange) ORC binding sites during helicase loading is shown. (B) Nucleotide frequency plots (aligned by MCM2-7 DH centre) and sequence logos (aligned by A-element) of the top and bottom 10% of origins identified by ChIP-Exo 5.0 sorted by replication timing[17]. Early origins produce a more prominent AT-skew than late origins and show a higher similarity to the standard A-element sequence. Areas covered by MCM2-7 DH are highlighted (green) and cover the switch of T- to A-rich strands. Nucleotide colour code as in (A). (C) The AT-skew at origins positions ORC (triangle) for head-to-head MCM2-7 double-hexamer formation. In presence of an AT-skew, ORC can bind to origins in a head-to-head positioning (green triangles), ideal for MCM2-7 DH deposition. When no skew is present, loading is unfavourable (red triangles). Sequences are for demonstration purposes only. For (a-b), source data is provided as a Source Data file.

a

description	sequence	Length (bp)	Motifs identified using FIMO motif scanning (*p*-values)		
			$1x10^{-4}$	$1x10^{-5}$	$1x10^{-6}$
Reuter et al.	AAAAAGAAATAATAAAAAAAAAAAAATAAAAA (WRWHDNDDNNNDHNWNWWDWAAAAAATAAAAA)	32	641	235	105
Chang et al. (complete)	ATGAAGATAAGTTAATAAAATATATAAAAATATATAAAAATAAGTATATAAAGA (DWNNHRRHWDDWDHRYMWHHHWDAHWWAAATVHHHAVHDNWHNDWWDADDHDDW)	54	253	53	13
Chang et al. (smaller)	(ANWWAAAT)	8	17	0	0
Chang et al. (degenerated)	(ANWWAAAN)	8	1	0	0

b

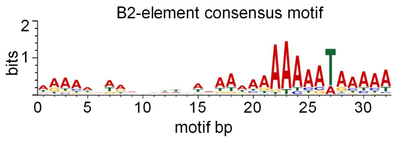

c

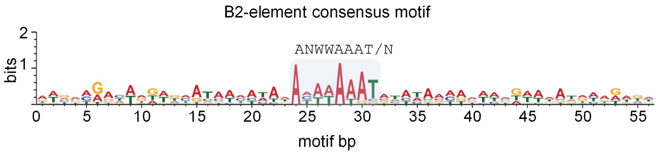

Figure S5: Comparison of identified B2-elements.

(A) Tabular overview of the identified B2-elements with description, sequence, length, and number of identified B2-elements given a minimal *p*-value for detection (motif occurrence), using 228 *ARS*-containing sequences as previously published[16]. Motif occurrence is defined as the probability of a random sequence of the same length as the motif matching the position of the sequence with as good or better of a score. Sequences in brackets represent the identified consensus motif in IUPAC nucleotide code. (B and C) Position weight matrix motifs of the different B2-elements. The consensus motif of the B2-element is shown for this study (B) and a previous study[16] (C). The light blue box highlights the minimal B2 consensus motif, as well as the degenerated version used in (A). Source data is provided as a Source Data file.

a

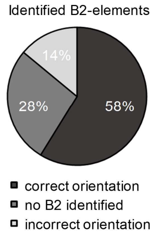

b

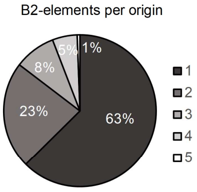

No. of B2 elements	No. of correctly orientated B2
1	114
2	41
3	15
4	10
5	1

c

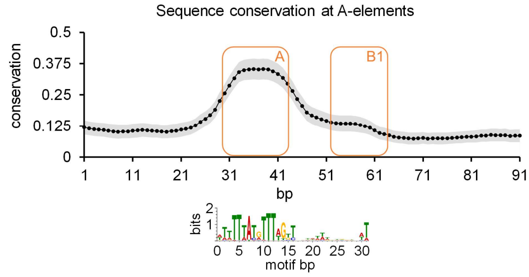

d

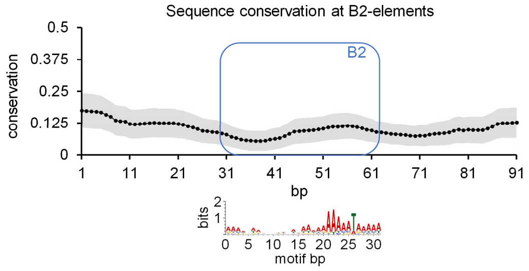

e

DNA feature	A-/B1- element	control	B2-element
Minor groove width	87.6%	0%	74.2%
Helical twist	74.9%	0%	74.2%
Propeller twist	88.5%	0%	80.7%
Roll	77.6%	0%	69%
Electrostatic potential	43.2%	0%	73.9%

f

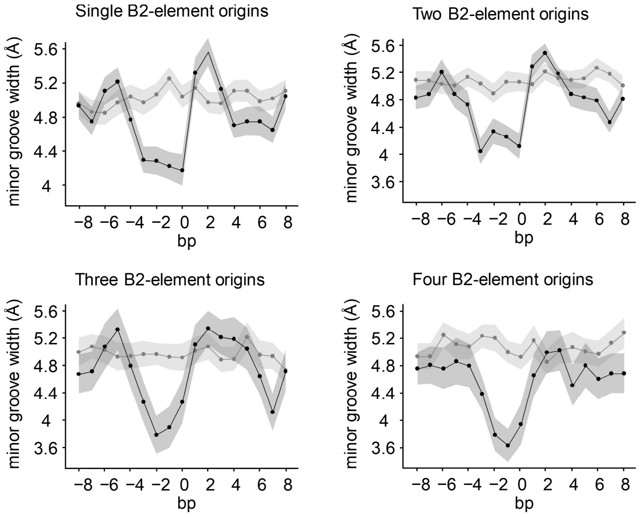

g

origin DNA element	Deformation energy (kcal/mol)
A- and B1-elements	147.1 ±0.7
B2-elements	152.8 ±0.9
controls	164.4 ±0.4

Figure S6: B2-element occurrence, conservation, and local DNA properties.

(A) Most origins have a correctly spaced B2-element. Pie chart showing the distribution of B2-element identification at all tested origins within 150 bp of the A-element. An incorrect orientation would result in an unfavourable rotational alignment of ORC, hindering second Mcm2-7 hexamer loading. (B) A single B2-element was identified at the majority of origins. Pie chart showing the distribution of B2-element occurrences at origins. (C and D) A-elements (C) but not B2-elements (D) are conserved amongst the *Saccharomyceta* clade. Evolutionary sequence scoring was calculated per bp of A- and B2-elements ±30 bp using phastcons7way (7 different *Saccharomyces* species[18]). Black dots represent the mean value of conservation at a given position with the surrounding 95% confidence interval in grey. A- and B1-elements are highlighted by orange boxes, B2-elements by blue boxes with a representation of the

underlying consensus motifs. (E) DNA is deformed at A- and B2-elements in *S. cerevisiae*. DNA shape changes relative to control regions for minor groove width, helical twist, propeller twist, roll (GB shape[19]), and electrostatic potential (phi; an approximation for steric impact on groove geometry[20]). Quantification to Figure 5F, G, and H. (F) Minor groove width changes can predict B2-element location at single and multiple B2-element origins. Visualised are the minor groove widths at the central thymidine of B2-elements (black) ±8 bp over control regions (grey, 500 bp downstream of B2-elements). Black dots represent the median value of the minor groove width at a given position with the surrounding 95% confidence interval in grey. (G) A- and B2-elements produce similar deformation energies in complex with ORC. Deformation energies for A- and B2-elements, as well as 1000 randomly generated sequences with the same GC-content were calculated based on the ORC-72 bp structure (PDB: 5ZR1[21]). Sequences were analysed with CURVES+[22] followed by conformational flexibility calculations using a multivariate Ising model[23] that incorporates relevant local effects (bimodality and nearest-neighbour coupling). Source data is provided as a Source Data file.

Reuter et al Figure S7

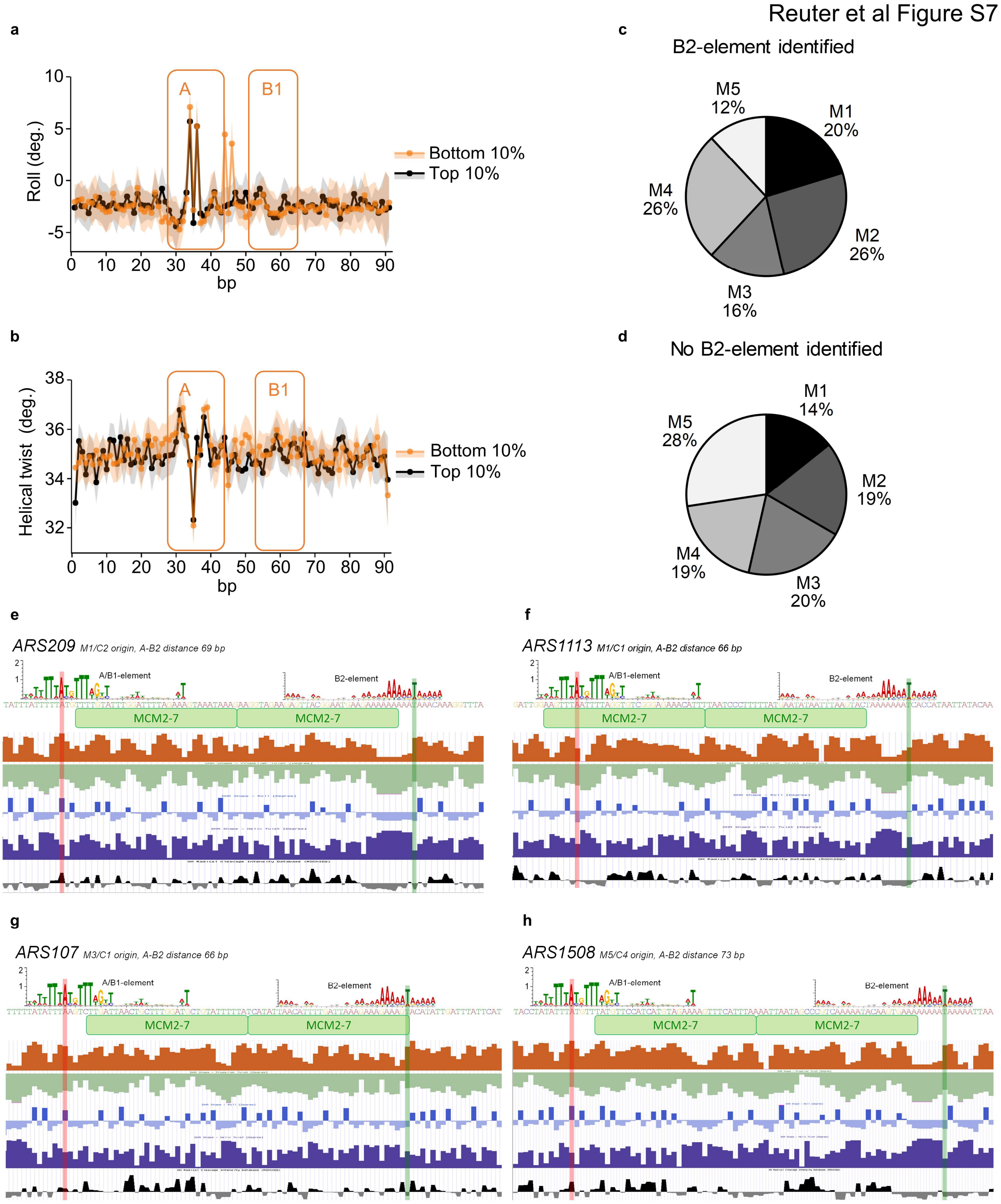

Figure S7: Organisation of the origin influences DNA licensing

DNA deformation at A-elements is not associated with origin licensing efficiency. Changes in roll (A) and helical twist (B) around A-elements of origins with identified B2-elements (n=181, each 10%) sorted by MCM2-7 loading are shown. Aligned A-elements of origins with most (black) and least (orange) MCM2-7 ChIP-Exo 5.0 reads are depicted ±30 bp surrounding the respective A-/B1-element (orange boxes). Individual dots represent the median deformation at a given position with the surrounding 95% confidence interval shown in black or orange. (C and D) Highly efficient origins (C, highest MCM2-7 DH signals, classes M1 and M2) cluster

with origins that contain a B2-element, while less efficient origins (D, classes M3-M5) cluster to origins without prominent B2-elements. Pie charts highlighting the distribution of origins (M1-M5) based on MCM2-7 DH signal in G1-phase (licensed origins). (E-H) Origins are compact structural DNA elements with the A-/B1-element and B2-element often covered by the MCM2-7 DH post DNA licensing. Positions and consensus motifs of A-/B1- and B2-elements are highlighted, as well as origin sequence and corresponding local DNA features (minor groove width, propeller twist, helix twist, roll, and OH radical cleavage intensities) at (E) *ARS209*, (F) *ARS1113*), (G) *ARS107*, and (H) *ARS1508*. Figures exported from Genome Browser (https://genome-euro.ucsc.edu/). Source data is provided as a Source Data file.

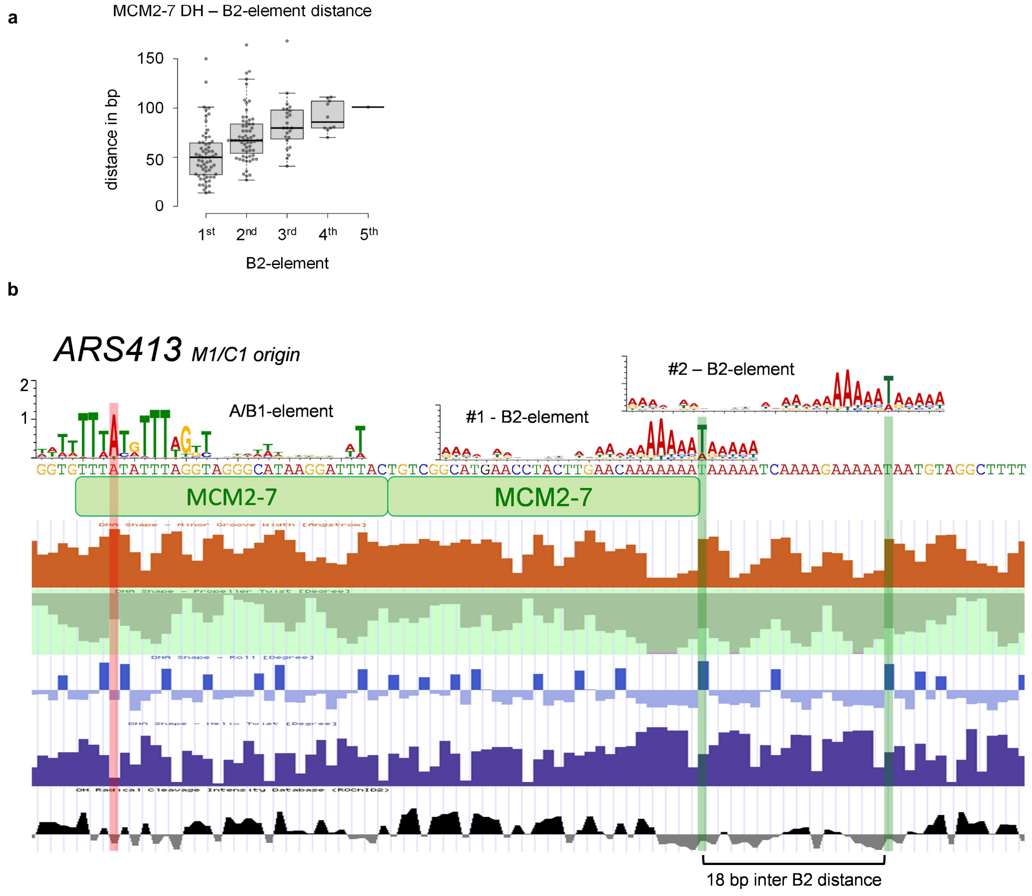

Figure S8: Multiple B2-elements are spaced in regular intervals.

(A) Origins with multiple, correctly orientated B2-elements have regularly-spaced B2-elements. Individual values, as well as the median, 1st, and 3rd quartiles, are shown. Whiskers extend to <1.5x IQR from 1st and 3rd quartiles. (B) *ARS413* is an exemplary origin with two consecutively spaced B2-elements. Positions and consensus motifs of A- and B2-elements are highlighted, as well as the origin sequence and corresponding local DNA features (from top to bottom: minor groove width, propeller twist, helix twist, roll, and OH radical cleavage intensities). Figures exported from Genome Browser (https://genome-euro.ucsc.edu/). For (a) data is provided as a Source Data file.

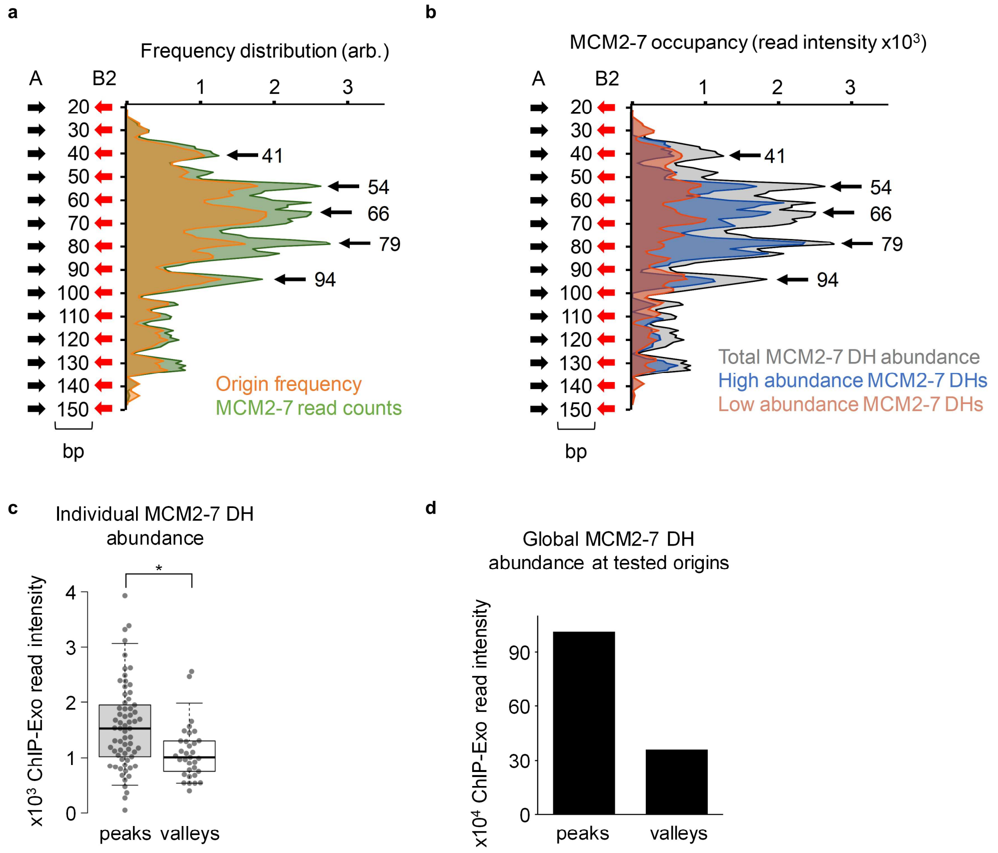

Figure S9: MCM2-7 DH abundance correlates with B2-element positioning.

(A) MCM2-7 DH abundance resembles A-/B2-element peak and valley clustering. Peak distances are indicated by black arrows and numbers. Areas are coloured accordingly: all origins with identified B2-elements (orange) and total MCM2-7 DH abundance (green). (B) Origins with high MCM2-7 DH abundance cluster to peak A-/B2-distances. Areas are coloured accordingly: total MCM2-7 DH abundance (grey), M1-M2 clustered origins (high MCM2-7 DH signal, blue), and M3-M5 clustered origins (lower and lowest MCM2-7 DH signal, red). (C) Individual peak origins load MCM2-7 DHs more efficiently than valley origins. Individual values, as well as the median, 1st, and 3rd quartiles, are shown. Whiskers extend to 5th and 95th percentile. The significance level was calculated using a two-sided Student's t-test (*: $p < 0.01$). (D) Peak origins show a higher global MCM2-7 DH abundance than valley origins. The sum of ChIP-Exo intensities of origins with peak- and valley-spaced B2-elements. Source data is provided as a Source Data file.

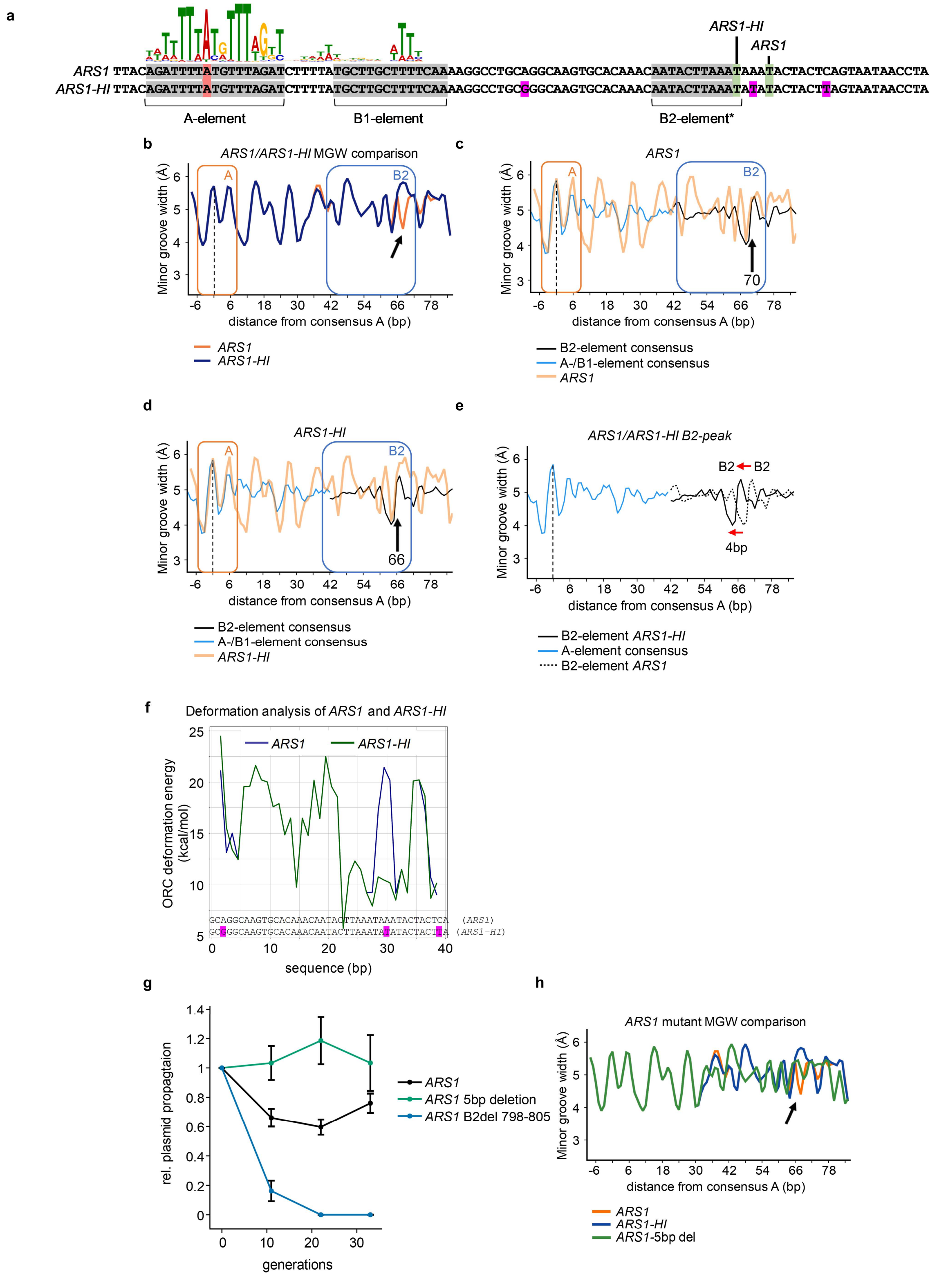

Figure S10: Modifying *ARS1* for optimal origin function *in vivo*.

(A) Sequence comparison between *ARS1* and *ARS1-HI* - the most efficient allele identified by mutARS-seq[24]. The canonical positions and compositions of the A-, B1, and B2-elements are marked (grey). B2-element* denotes the previously described location and motif. Highlighted

are key conserved thymidines used to map B2-elements (green, consensus thymindine as described in[24]), the consensus adenine of the ACS (red), and sequence differences (purple). (B) to (E) Minor groove width (MGW) plot of *ARS1* and *ARS1-H1* from (A). Positions of the A- (orange) and B2-elements (blue) are indicated as well as the central adenine of the A-element (black dashed line). (B) Comparison of individual minor groove width changes between *ARS1* (C) and *ARS1-HI* (D). The consensus A- (blue) and B2-element (black) distortion of the MGW are indicated at observed sites. The location of the conserved thymidine of the B2-element is indicated (arrow) highlighting its distance from the central adenine of the A-element. (E) Consensus B2-element location differences between *ARS1* (black dotted line) and *ARS1-HI* (black line). The consensus A-element distortion (blue) of the MGW is indicated at observed sites. The red arrow highlights the shift of four bases from *ARS1* to *ARS1-HI*, moving it into a peak A-B2-distance for efficient helicase loading (compare to Figure 6 and S7). (F) The B2-element of *ARS1-HI* is more flexible than the canonical *ARS1* B2-element. Deformation energies for the B2-elements of *ARS1* and *ARS1-HI* were calculated based on the ORC-72 bp structure in a 41 bp window (PDB: 5ZR1[21]). Sequences were analysed with CURVES+[22] followed by conformational flexibility calculations using a multivariate Ising model[23] that incorporates relevant local effects (bimodality and nearest-neighbour coupling). (G) Reducing the A-B2-element distance renders *ARS1* a more efficient origin. Plasmid propagation assays were performed with *CEN* vectors containing indicated ARSs: *ARS1*, *ARS1*_del5, and *ARS1*_B2-(798-805)[25]. Plotted is the relative plasmid propagation as the average of three biological replicates and the standard error. (H) *ARS1*_del5 resembles the *ARS1-HI* DNA structure. Minor groove width plot overlay of *ARS1* (orange), *ARS1-HI* (blue), and *ARS1*_del5 (green). The arrow (black) indicates the shifted B2-element. For (a-e) and (g-h), source data is provided as a Source Data file.

SUPPLEMENTARY TABLES

Supplementary table 1. Yeast strains

Genotype	Identifier	Source
W303, *MATa, ade2-1, ura3-1, his3-11,15, trp1-1, leu2-3, 112, can1-100*	YC160	Rothstein, R.[26]
W303, *MAT*a, *ade2-1, trp1-1, can1-100, leu2-3,112, his3-11,15, ura3, RAD5+*	YC510	Andrei Chabes
YC160*, MCM4::MCM4-5xFlag::hphNT*	YC779	This study
YC160, *ORC2::ORC2-5xFlag::hphNT*	YC795	This study
YC779, *pMet::cdc20::TRP1*	YC840	This study
YC795, *pMet::cdc20::TRP1*	YC842	This study
YB44, W303, *MAT*a, *ade2-1, ura3-1, his3-11,15, trp1-1, leu2-3,112 can1-100, CDC6::cdc6-1*	YC819	Bruce Stillman[27]
YB564, W303, *MAT*a, *ade2-1, ura3-1, his3-11,15, trp1-1, leu2-3,112 can1-100, CDC46::cdc46-1*	YC823	Bruce Stillman[27]
YC819, *ORC2::ORC2-5xFLAG::hphNT*	YC894	This study
YC823, *ORC2::ORC2-5xFLAG::hphNT*	YC895	This study

Supplementary table 2. Plasmids

Details	Identifier	Source
pKL255 (5xFLAG-hphNT)	pCS762	Labib, K.
pMet-*cdc20-TRP*	pCS1258	Aragon, L.
pARS1/WTA	pCS177	Bruce Stillman[25]
pARS1/798-805 (B2-)	pCS174	Bruce Stillman[25]
**pARS1_del5**	pCS1414	This study

Supplementary table 3. Oligonucleotides

Sequence 5’-3’	Identifier	Source
**qPCR oligonucleotides**		
AGCCTTCTTTGGAGCTCAAG	ARS305_fw	This study
TTTGAGGAATTTCTTTTGAAGAG	ARS305_rev	This study
AACTTGGCCTTATGTAGAATTTCTT	ARS305_+9fw	This study
AGCAATTCCACCGACCATAC	ARS305_+9rev	This study
CTCATCATCATCCCCGGTA	ARS501_fw	This study
CGTACACTAGCCCGTTGAGGT	ARS501_rev	This study
GTGGTGATATAAACACTACATTCGC	ARS607_fw	This study
TCCATAATATTTACCTTACGCTGGG	ARS607_rev	This study
GCGTGTGTTGGTTACTTAGGTT	ARS1021_fw	This study
GGCTCTGTTTTGTTTATTTCCAGAT	ARS1021_rev	This study
GCCAGTAACCCATGTGTGAAG	ARS307_fw	This study
ACTTTCTTGTGTGGGCTGCT	ARS307_rev	This study
AGTTCTTGGACAAGATTGGATACAT	ARS1429_fw	This study
TTACCTTTCAATGCGGCACG	ARS1429_rev	This study
TCTTGCCCACTTCTAAGCTGAT	MAT_fw	This study
TGCATCCCAAACAAAACCCAG	MAT_rev	This study
ACCCAGCTTATTGCTCTGGT	YPC1_fw	This study
GTACATGTCCCGAATTAGCTAACAA	YPC1_rev	This study
GCTCAGGGTTCATCATGCCA	FDH1_fw	This study
CCGCCCAGCCTAGAAAGTC	FDH1_rev	This study
TGTGTATGCGTTCCGAACTT	YPT7_fw	This study
AGCCAATTTATCCTGTCGTTGA	YPT7_rev	This study
CAAAATGCGAGGTGTGGGTG	DPB11_fw	This study
GAGGAAATCGGAGAGGCAGA	DPB11_rev	This study
**Mutagenesis oligonucleotides**		
GCTTGCTTTTCAAAAGCAGGCAAGTGCACAAAC	ARS1_5bp_deletion_rev	This study
GTTTGTGCACTTGCCTGCTTTTGAAAAGCAAGC	ARS1_5bp_deletion_fw	This study

**Genomic integration oligonucleotides**		
GTCCTTGGCGAGGGTGTAAGGAGATCAGTTCGCCTGAA TAACCGTGTCCGTACGCTGCAGGTCGAC	MCM4-tag_fw	This study
TAATTAGTATTTATTAATTGTTACGCAGGGAATGATTGTA GTAGACAGCAATCGATGAATTCGAGCTCG	MCM4-tag_rev	This study
TATGCGGAACTTGAAAAACTTCTGAAAACCGTTTTAAATA CTCTACGTACGCTGCAGGTCGAC	ORC2-tag_fw	This study
AGCTAGCAAGCCTAGTACTATTACAATTGTTCGTGATATG TATACATTTAATCGATGAATTCGAGCTCG	ORC2-tag_rev	This study
**ChIP-Exo oligonucleotides**		
5-Phos CAA GCA GAA GAC GGC ATA CGA GAT TCGCCTTA GTG ACT GGA GTT CAG ACG TGT GCT CTT CCG ATC T	ExA2_iNN_701	This study
5-Phos CAA GCA GAA GAC GGC ATA CGA GAT CTAGTACG GTG ACT GGA GTT CAG ACG TGT GCT CTT CCG ATC T	ExA2_iNN_702	This study
5-Phos CAA GCA GAA GAC GGC ATA CGA GAT TTCTGCCT GTG ACT GGA GTT CAG ACG TGT GCT CTT CCG ATC T	ExA2_iNN_703	This study
5-Phos CAA GCA GAA GAC GGC ATA CGA GAT GCTCAGGA GTG ACT GGA GTT CAG ACG TGT GCT CTT CCG ATC T	ExA2_iNN_704	This study
5-Phos CAA GCA GAA GAC GGC ATA CGA GAT AGGAGTCC GTG ACT GGA GTT CAG ACG TGT GCT CTT CCG ATC T	ExA2_iNN_705	This study
5-Phos CAA GCA GAA GAC GGC ATA CGA GAT CATGCCTA GTG ACT GGA GTT CAG ACG TGT GCT CTT CCG ATC T	ExA2_iNN_706	This study
5-Phos CAA GCA GAA GAC GGC ATA CGA GAT GTAGAGAG GTG ACT GGA GTT CAG ACG TGT GCT CTT CCG ATC T	ExA2_iNN_707	This study
5-Phos CAA GCA GAA GAC GGC ATA CGA GAT CCTCTCTG GTG ACT GGA GTT CAG ACG TGT GCT CTT CCG ATC T	ExA2_iNN_708	This study
AAT GAT ACG GCG ACC ACC GAG ATC TAC ACT CTT TCC CTA CAC GAC GCT CTT CCG ATC T	ExA1-58	Rossi[28]
GAT CGG AAG AGC ACA CGT CTG AAC TCC AGT CAC	ExA2B	Rossi[28]
NNN NNA GAT CGG AAG AGC G	ExA1-SSL_N5	Rossi[28]
AAT GAT ACG GCG ACC ACC	P1.3	Rossi[28]
CAA GCA GAA GAC GGC ATA CGA G	P2.1	Rossi[28]

Supplementary table 4. DNA sequences for DNA deformation analysis

B2-element prediction	DNA sequence
ARS1	GCAGGCAAGTGCACAAACAATACTTAAATAAATACTACTCA
ARS1-HI[24]	GCGGGCAAGTGCACAAACAATACTTAAATATATACTACTTA

SUPPLEMENTARY REFERENCES

1. Muller, P. et al. The conserved bromo-adjacent homology domain of yeast Orc1 functions in the selection of DNA replication origins within chromatin. *Genes Dev* **24**, 1418-33 (2010).
2. Hoggard, T., Hollatz, A.J., Cherney, R.E., Seman, M.R. & Fox, C.A. The Fkh1 Forkhead associated domain promotes ORC binding to a subset of DNA replication origins in budding yeast. *Nucleic Acids Res* **49**, 10207-10220 (2021).
3. Sanchez, H. et al. DNA replication origins retain mobile licensing proteins. *Nat Commun* **12**, 1908 (2021).
4. Zhang, A., Friedman, L.J., Gelles, J. & Bell, S.P. Changing protein-DNA interactions promote ORC binding-site exchange during replication origin licensing. *Proc Natl Acad Sci U S A* **120**, e2305556120 (2023).
5. Fox, C.A., Loo, S., Rivier, D.H., Foss, M.A. & Rine, J. A transcriptional silencer as a specialized origin of replication that establishes functional domains of chromatin. *Cold Spring Harb Symp Quant Biol* **58**, 443-55 (1993).
6. Spellman, P.T. et al. Comprehensive identification of cell cycle-regulated genes of the yeast Saccharomyces cerevisiae by microarray hybridization. *Mol Biol Cell* **9**, 3273-97 (1998).
7. Miotto, B., Ji, Z. & Struhl, K. Selectivity of ORC binding sites and the relation to replication timing, fragile sites, and deletions in cancers. *Proc Natl Acad Sci U S A* **113**, E4810-9 (2016).
8. Shor, E. et al. The origin recognition complex interacts with a subset of metabolic genes tightly linked to origins of replication. *PLoS Genet* **5**, e1000755 (2009).
9. Lee, C.S.K. et al. Humanizing the yeast origin recognition complex. *Nat Commun* **12**, 33 (2021).
10. Yuan, Z. et al. Structural basis of Mcm2-7 replicative helicase loading by ORC-Cdc6 and Cdt1. *Nat Struct Mol Biol* **24**, 316-324 (2017).
11. Remus, D. et al. Concerted loading of Mcm2-7 double hexamers around DNA during DNA replication origin licensing. *Cell* **139**, 719-30 (2009).
12. Chacin, E. et al. Establishment and function of chromatin organization at replication origins. *Nature* **616**, 836-842 (2023).
13. Hu, Y. et al. Evolution of DNA replication origin specification and gene silencing mechanisms. *Nat Commun* **11**, 5175 (2020).
14. Hemerly, A.S., Prasanth, S.G., Siddiqui, K. & Stillman, B. Orc1 controls centriole and centrosome copy number in human cells. *Science* **323**, 789-93 (2009).
15. Nieduszynski, C.A., Hiraga, S., Ak, P., Benham, C.J. & Donaldson, A.D. OriDB: a DNA replication origin database. *Nucleic Acids Res* **35**, D40-6 (2007).
16. Chang, F. et al. High-resolution analysis of four efficient yeast replication origins reveals new insights into the ORC and putative MCM binding elements. *Nucleic Acids Res* **39**, 6523-35 (2011).
17. Yabuki, N., Terashima, H. & Kitada, K. Mapping of early firing origins on a replication profile of budding yeast. *Genes Cells* **7**, 781-9 (2002).
18. Ramani, R., Krumholz, K., Huang, Y.F. & Siepel, A. PhastWeb: a web interface for evolutionary conservation scoring of multiple sequence alignments using phastCons and phyloP. *Bioinformatics* **35**, 2320-2322 (2019).

19. Chiu, T.P. et al. GBshape: a genome browser database for DNA shape annotations. *Nucleic Acids Res* **43**, D103-9 (2015).
20. Chiu, T.P., Rao, S., Mann, R.S., Honig, B. & Rohs, R. Genome-wide prediction of minor-groove electrostatic potential enables biophysical modeling of protein-DNA binding. *Nucleic Acids Res* **45**, 12565-12576 (2017).
21. Li, N. et al. Structure of the origin recognition complex bound to DNA replication origin. *Nature* **559**, 217-222 (2018).
22. Lavery, R., Moakher, M., Maddocks, J.H., Petkeviciute, D. & Zakrzewska, K. Conformational analysis of nucleic acids revisited: Curves+. *Nucleic Acids Res* **37**, 5917-29 (2009).
23. Liebl, K. & Zacharias, M. Accurate modeling of DNA conformational flexibility by a multivariate Ising model. *Proc Natl Acad Sci U S A* **118**(2021).
24. Liachko, I., Youngblood, R.A., Keich, U. & Dunham, M.J. High-resolution mapping, characterization, and optimization of autonomously replicating sequences in yeast. *Genome Res* **23**, 698-704 (2013).
25. Marahrens, Y. & Stillman, B. Replicator dominance in a eukaryotic chromosome. *EMBO J* **13**, 3395-400 (1994).
26. Thomas, B.J. & Rothstein, R. Elevated recombination rates in transcriptionally active DNA. *Cell* **56**, 619-30 (1989).
27. Liang, C., Weinreich, M. & Stillman, B. ORC and Cdc6p interact and determine the frequency of initiation of DNA replication in the genome. *Cell* **81**, 667-76 (1995).
28. Rossi, M.J., Lai, W.K.M. & Pugh, B.F. Simplified ChIP-exo assays. *Nat Commun* **9**, 2842 (2018).
