# Supplementary material for: MCM2-7 loading-dependent ORC release ensures genome-wide origin licensing
Source: Nat Commun. 2024 Aug 24;15:7306. doi: 10.1038/s41467-024-51538-9 (PMC11344781; doi:10.1038/s41467-024-51538-9)
Supplement: Supplementary file 3 — Description of Additional Supplementary Files [file 41467_2024_51538_MOESM3_ESM.pdf]

Description of Additional Supplementary Files

File Name: Supplementary Data 1
Description: Genomic location of origin elements, non-origin binding sites and ORC/MCM2-7 binding sites.
