# Supplementary material for: MCM2-7 loading-dependent ORC release ensures genome-wide origin licensing
Source: Nat Commun. 2024 Aug 24;15:7306. doi: 10.1038/s41467-024-51538-9 (PMC11344781; doi:10.1038/s41467-024-51538-9)
Supplement: Supplementary file 5 — Reporting Summary [file 41467_2024_51538_MOESM5_ESM.pdf]

nature portfolio

Corresponding author(s): L. Maximilian Reuter and Christian Speck

Last updated by author(s): May 7, 2024

Reporting Summary

Nature Portfolio wishes to improve the reproducibility of the work that we publish. This form provides structure for consistency and transparency in reporting. For further information on Nature Portfolio policies, see our Editorial Policies and the Editorial Policy Checklist.

Statistics

For all statistical analyses, confirm that the following items are present in the figure legend, table legend, main text, or Methods section.

n/a	Confirmed	
☐	☒	The exact sample size (*n*) for each experimental group/condition, given as a discrete number and unit of measurement
☐	☒	A statement on whether measurements were taken from distinct samples or whether the same sample was measured repeatedly
☐	☒	The statistical test(s) used AND whether they are one- or two-sided<br>*Only common tests should be described solely by name; describe more complex techniques in the Methods section.*
☒	☐	A description of all covariates tested
☐	☒	A description of any assumptions or corrections, such as tests of normality and adjustment for multiple comparisons
☐	☒	A full description of the statistical parameters including central tendency (e.g. means) or other basic estimates (e.g. regression coefficient) AND variation (e.g. standard deviation) or associated estimates of uncertainty (e.g. confidence intervals)
☐	☒	For null hypothesis testing, the test statistic (e.g. *F*, *t*, *r*) with confidence intervals, effect sizes, degrees of freedom and *P* value noted<br>*Give P values as exact values whenever suitable.*
☒	☐	For Bayesian analysis, information on the choice of priors and Markov chain Monte Carlo settings
☒	☐	For hierarchical and complex designs, identification of the appropriate level for tests and full reporting of outcomes
☒	☐	Estimates of effect sizes (e.g. Cohen's *d*, Pearson's *r*), indicating how they were calculated

Our web collection on statistics for biologists contains articles on many of the points above.

Software and code

Policy information about availability of computer code

Data collection	For ChIP-qPCR, data collection was perfomed on a Biorad CF96 qPCR cycler and data was exported from the machine with Biorad CFX Manager (ver. 3.1)<br>For ChIP-Seq, data collection was performed on a Illumina HiSeq 2500 and data was processed as described in the M&M section and below.<br>For ChIP-Exo, data collection was performed on a Illumina HiSeq 2500 and data was processed as described in the M&M section and below.
Data analysis	ChIP-qPCR: CFX Maestro 2.1,<br>Sequencing data:<br>FastQC (ver. 0.11.5), bcl2fastq2_2.20.0, ChIPexoQual (https://welch16.github.io/ChIPexoQual/index.html), Bowtie2 aligner (ver. 2.3.5), deeptools (ver. 3.2.1), bedtools (version 2.27.1), WebLogo 2.8.2 (https://weblogo.berkeley.edu/), MEME Suite (ver. 5.3.0), ChExMix (ver. 0.45),ChIPpeakAnno (ver. 3.38)<br>ChAsE: Chromatin Analysis and Exploration Tool (https://github.com/hyounesy/ChAsE),<br>Code for the Ising model computations (https://github.com/XiPacha/Ising-model),<br>CURVES+ (v3.0 +, http://curvesplus.bsc.es/).

For manuscripts utilizing custom algorithms or software that are central to the research but not yet described in published literature, software must be made available to editors and reviewers. We strongly encourage code deposition in a community repository (e.g. GitHub). See the Nature Portfolio guidelines for submitting code & software for further information.

Data

Policy information about availability of data

All manuscripts must include a data availability statement. This statement should provide the following information, where applicable:

- Accession codes, unique identifiers, or web links for publicly available datasets
- A description of any restrictions on data availability
- For clinical datasets or third party data, please ensure that the statement adheres to our policy

ChIP-Exo data were deposited into the Gene Expression Omnibus database under accession number GSE240779 and are available at the following URL: https://www.ncbi.nlm.nih.gov/geo/query/acc.cgi?acc=GSE240779. Code for the Ising model computations is available at: https://github.com/XiPacha/Ising-model.

Databases/datasets used in the study:
Reference genome: SacCer3 (https://www.ncbi.nlm.nih.gov/datasets/genome/GCF_000146045.2/)
Origin location data: OriDB (https://cerevisiae.oridb.org/index.php)
Replication timing data: Extracted from OriDB at https://cerevisiae.oridb.org/data_output.php?main=sc_repl_data&table=Raghu2001_Trep&ext_format=WIG;&format=tab (Raghuraman et al., 2001) and https://cerevisiae.oridb.org/data_output.php?main=sc_repl_data&table=Yabuki2002_Trep&ext_format=WIG;&format=tab (Yabuki et al., 2002)
DNA properties data: GBshape (http://rohsdb.cmb.usc.edu/GBshape)
Electrostatic potential data: DNAphi (https://rohslab.usc.edu/DNAphi/index.html)
Sequence conservation data: PhastWeb (http://compgen.cshl.edu/phastweb)

Coordinate files for the location of MCM2-7 DHs, ORC, A- and B2-elements are available in the supplemenatry information.

Research involving human participants, their data, or biological material

Policy information about studies with human participants or human data. See also policy information about sex, gender (identity/presentation), and sexual orientation and race, ethnicity and racism.

Reporting on sex and gender	n/a
Reporting on race, ethnicity, or other socially relevant groupings	n/a
Population characteristics	n/a
Recruitment	n/a
Ethics oversight	n/a

Note that full information on the approval of the study protocol must also be provided in the manuscript.

Field-specific reporting

Please select the one below that is the best fit for your research. If you are not sure, read the appropriate sections before making your selection.

☒ Life sciences ☐ Behavioural & social sciences ☐ Ecological, evolutionary & environmental sciences

For a reference copy of the document with all sections, see nature.com/documents/nr-reporting-summary-flat.pdf

Life sciences study design

All studies must disclose on these points even when the disclosure is negative.

Sample size	No statistical methods were used to predetermine sample size. Sample sizes were chosen for adequate power (genome coverage) to detect meaningful differences and was guided by standards in the field.
Data exclusions	No data were excluded
Replication	Conclusions in this study, which are based on generated data were verified by at least two individual biological replicates and/or by merging of at least two biologically independent replicates.
Randomization	Samples were not randomized, as no allocation into experimental groups was performed.
Blinding	The investigators were not blinded during the experimental nor assessment stages due to the intrinsic unbiased nature of the respective assays/analyses and no group allocation was involved in this study.

Reporting for specific materials, systems and methods

We require information from authors about some types of materials, experimental systems and methods used in many studies. Here, indicate whether each material, system or method listed is relevant to your study. If you are not sure if a list item applies to your research, read the appropriate section before selecting a response.

Materials & experimental systems

n/a	Involved in the study
☐	☒ Antibodies
☒	☐ Eukaryotic cell lines
☒	☐ Palaeontology and archaeology
☒	☐ Animals and other organisms
☒	☐ Clinical data
☒	☐ Dual use research of concern
☒	☐ Plants

Methods

n/a	Involved in the study
☐	☒ ChIP-seq
☒	☐ Flow cytometry
☒	☐ MRI-based neuroimaging

Antibodies

Antibodies used: anti-FLAG, Sigma, F-1804, 1/300 dilution (various lot #)

Validation: The anti-Flag antibody was validated by Western Blotting against FLAG protein and by ChIP-qPCR at on and off target sites of several FLAG-tagged proteins
https://www.sigmaaldrich.com/catalog/product/sigma/f1804?lang=de&region=GB

Plants

Seed stocks: *Report on the source of all seed stocks or other plant material used. If applicable, state the seed stock centre and catalogue number. If plant specimens were collected from the field, describe the collection location, date and sampling procedures.*

Novel plant genotypes: *Describe the methods by which all novel plant genotypes were produced. This includes those generated by transgenic approaches, gene editing, chemical/radiation-based mutagenesis and hybridization. For transgenic lines, describe the transformation method, the number of independent lines analyzed and the generation upon which experiments were performed. For gene-edited lines, describe the editor used, the endogenous sequence targeted for editing, the targeting guide RNA sequence (if applicable) and how the editor was applied.*

Authentication: *Describe any authentication procedures for each seed stock used or novel genotype generated. Describe any experiments used to assess the effect of a mutation and, where applicable, how potential secondary effects (e.g. second site T-DNA insertions, mosiacism, off-target gene editing) were examined.*

ChIP-seq

Data deposition

☒ Confirm that both raw and final processed data have been deposited in a public database such as GEO.

☒ Confirm that you have deposited or provided access to graph files (e.g. BED files) for the called peaks.

Data access links
May remain private before publication.

https://www.ncbi.nlm.nih.gov/geo/query/acc.cgi?acc=GSE240779

Files in database submission

IP_795_G1_37_1_S8_L001_R1_001.fastq.gz
IP_795_G1_37_2_S20_L001_R1_001.fastq.gz
IP_795_G1_37_3_S32_L001_R1_001.fastq.gz
IP_795_G2_37_1_S7_L001_R1_001.fastq.gz
IP_795_G2_37_2_S19_L001_R1_001.fastq.gz
IP_795_G2_37_3_S31_L001_R1_001.fastq.gz
IP_894_G1_37_1_S10_L001_R1_001.fastq.gz
IP_894_G1_37_2_S22_L001_R1_001.fastq.gz
IP_894_G1_37_3_S34_L001_R1_001.fastq.gz
IP_894_G2_37_1_S9_L001_R1_001.fastq.gz
IP_894_G2_37_2_S21_L001_R1_001.fastq.gz
IP_894_G2_37_3_S33_L001_R1_001.fastq.gz
IP_895_G1_37_1_S12_L001_R1_001.fastq.gz
IP_895_G1_37_2_S24_L001_R1_001.fastq.gz
IP_895_G1_37_3_S36_L001_R1_001.fastq.gz
IP_895_G2_37_1_S11_L001_R1_001.fastq.gz

IP_895_G2_37_2_S23_L001_R1_001.fastq.gz
IP_895_G2_37_3_S35_L001_R1_001.fastq.gz
Input_795_G1_37_1_S2_L001_R1_001.fastq.gz
Input_795_G1_37_2_S14_L001_R1_001.fastq.gz
Input_795_G1_37_3_S26_L001_R1_001.fastq.gz
Input_795_G2_37_1_S1_L001_R1_001.fastq.gz
Input_795_G2_37_2_S13_L001_R1_001.fastq.gz
Input_795_G2_37_3_S25_L001_R1_001.fastq.gz
Input_894_G1_37_1_S4_L001_R1_001.fastq.gz
Input_894_G1_37_2_S16_L001_R1_001.fastq.gz
Input_894_G1_37_3_S28_L001_R1_001.fastq.gz
Input_894_G2_37_1_S3_L001_R1_001.fastq.gz
Input_894_G2_37_2_S15_L001_R1_001.fastq.gz
Input_894_G2_37_3_S27_L001_R1_001.fastq.gz
Input_895_G1_37_1_S6_L001_R1_001.fastq.gz
ORC2_24min_1_S45_L005_R1_001.fastq.gz
ORC2_24min_2_S46_L005_R1_001.fastq.gz
Input_895_G1_37_2_S18_L001_R1_001.fastq.gz
Mcm4wt_G1_1_R1_001.fastq.gz
Mcm4wt_G1_2_R1_001.fastq.gz
Orc2wt_G1_1_R1_001.fastq.gz
Orc2wt_G1_2_R1_001.fastq.gz
Orc2wt_G2_1_R1_001.fastq.gz
Orc2wt_G2_2_R1_001.fastq.gz
Input_895_G1_37_3_S30_L001_R1_001.fastq.gz
Input_895_G2_37_1_S5_L001_R1_001.fastq.gz
Input_895_G2_37_2_S17_L001_R1_001.fastq.gz
Input_895_G2_37_3_S29_L001_R1_001.fastq.gz

Genome browser session
(e.g. UCSC)

no longer applicable

Methodology

Replicates

All samples for a given ChIP-Exo experiment were analyzed by ChexMix individually. The resulting binding events for each individual replicate sample can be found at GEO. --standard binding event reporting mode was used which report events that pass significance threshold in condition as a whole (Binomial, 1.5-fold change, q<0.01). In addition, ChIPexoQual, a Bioconductor package was used to perform ChIP-exo specific quality control. When ChExAlign was used, standard parameters were applied.
All samples for a given ChIP-Seq experiment were analyzed individually. Standard procedures for alignment and quality control were applied (see bleow).

Sequencing depth

Typical read depth: 3 million single end reads (minimum read length 40). A minimum of 1 million mapped reads were available for the analysis of particular sample. We determined the sequencing depth to be sufficient based on previously reported literature (M&M Ref 2).

Antibodies

See antibody section.

Peak calling parameters

Read mapping was performed using Bowtie2 (v 2.3.5) using default parameters. ChexMix version 0.45 was run with following custom settings --kldivergencethres -3 or -10 --nomotifs. We also used –exclude option to exclude blacklist regions that includes tRNA genes and the rDNA locus.

Data quality

All samples were evaluated for quality using ChIPexoQual, a Bioconductor package designed specifically for ChIP-exo data. The package generates several diagnostic plots and summary measures that enable assessing enrichment and library complexity. Raw sequence quality was also assessed using FastQC, a popular quality control tool for high throughput sequence data.

Software

Read mapping was performed using Bowtie2 (M&M Ref 7). ChIP-exo specific quality evaluation was performed using ChIPexoQual package (M&M Ref 6). Peak locations were obtained using ChExMix software (M&M Ref 12,13). Raw sequence quality was also assessed using FastQC (M&M Ref 5), a popular quality control tool for high throughput sequence data.
